# Microenvironmental barriers to healing in osteoradionecrosis and opportunities for regenerative intervention

**DOI:** 10.1016/j.bioactmat.2026.09.020

**Published:** 2026-10-13

**Authors:** Aleyna E. La Croix, Maria Eduarda Torres Gouveia, Peter M. Bertone, Neva M. Nemanic, Chelsea S. Bahney, Katherine R. Hixon

**Affiliations:** a Thayer School of Engineering, Dartmouth College, Hanover, NH, United States; b The Orthopaedic Trauma Institute, University of California, San Francisco (UCSF), San Francisco, CA, United States; c Center for Regenerative and Personalized Medicine, Steadman Philippon Research Institute, Vail, CO, United States; d Geisel School of Medicine, Dartmouth College, Hanover, NH, United States

**Keywords:** Osteoradionecrosis, Radiotherapy-induced senescence, Bone tissue engineering, Regenerative biomaterials, Mandibular reconstruction, Senotherapeutics, Vascularized bone healing

## Abstract

Osteoradionecrosis (ORN) is a debilitating sequela of radiotherapy (RT) for head and neck cancer (HNC), characterized by persistent bone exposure or radiographic non-viability and profound tissue repair failure. Despite advances in radiation delivery and clinical management, ORN remains challenging to treat, with therapeutic strategies largely focused on symptom control, infection mitigation, or surgical reconstruction rather than restoration of regenerative capacity. Emerging evidence suggests that radiation-induced cellular senescence represents a central and underrecognized driver of ORN pathophysiology. Ionizing radiation disrupts the bone microenvironment through sustained vascular injury, impaired osteogenic-angiogenic coupling, chronic inflammation, and the accumulation of senescent bone-resident and stromal cells that secrete a pro-inflammatory senescence-associated secretory phenotype (SASP). Together, these changes create a hostile niche that suppresses bone remodeling and wound healing long after radiation exposure. This review synthesizes current understanding of radiation-induced injury to the bone microenvironment, critically evaluates existing classification systems and treatment strategies for ORN, and examines emerging regenerative approaches. We further propose senotherapeutic strategies targeting radiation-induced cellular senescence as a complementary and potentially transformative avenue to restore tissue repair capacity. By integrating regenerative biomaterials with targeted modulation of senescence, future therapies may move beyond palliation toward true biological resolution of ORN. Biomaterial delivery systems (e.g., injectable hydrogels, mineral composite scaffolds, and cryogel platforms) are emerging as promising vehicles for spatiotemporally controlled senotherapeutic delivery within the irradiated bone niche.

## Highlights

• Radiation-induced senescence is a central, underrecognized driver of ORN.
• SASP signaling sustains a hostile, non-regenerative bone microenvironment.
• Cell-specific radiation responses disrupt osteogenic-angiogenic coupling.
• Scaffold-based biomaterials enable localized senotherapeutic delivery.
• Combining senolytics with regenerative scaffolds may resolve ORN biologically.

## Graphical abstract

**Figure:**
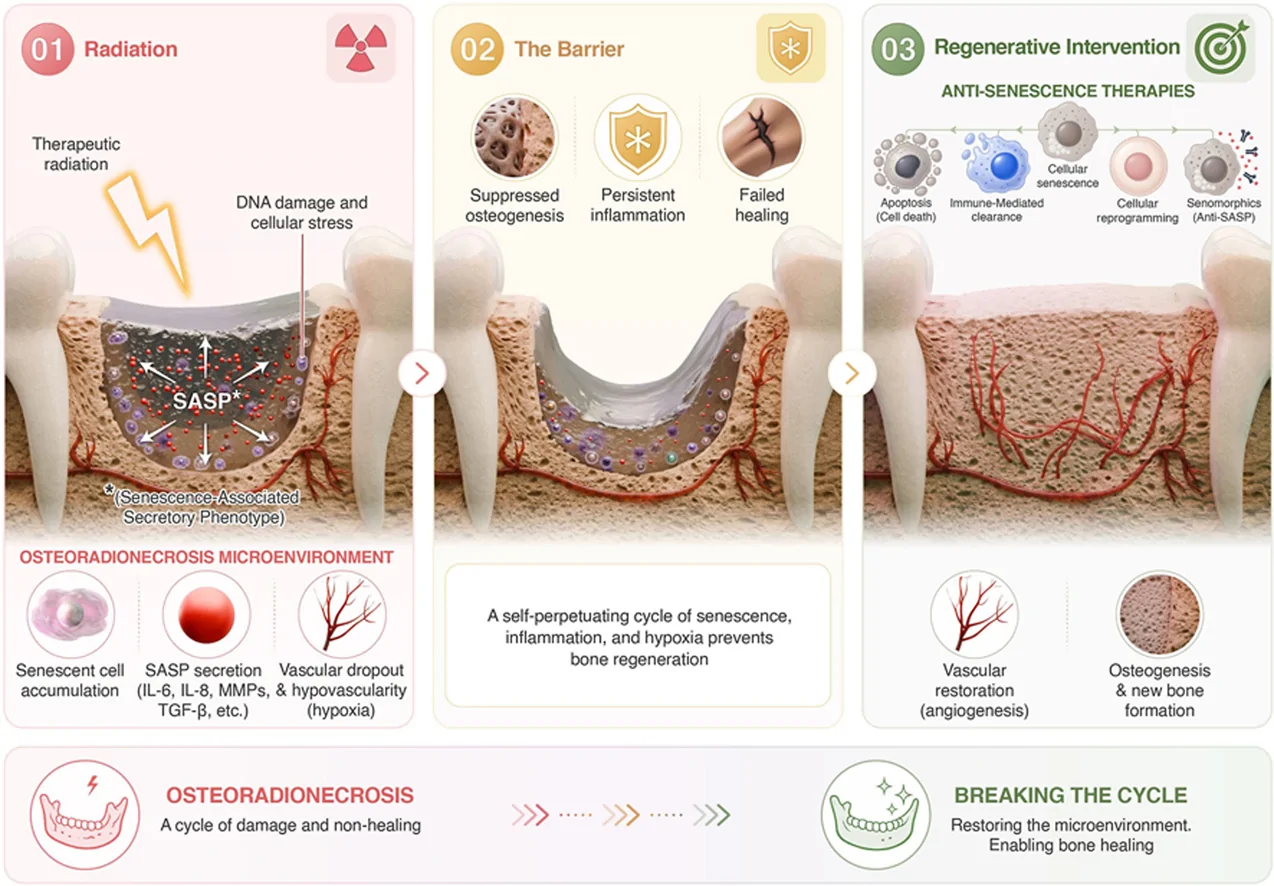

## 1 Introduction

Global cancer incidence is projected to rise to 28.4 million cases by 2040, representing a 47% increase from 2020, with a disproportionately higher burden in transitioning countries [1]. Among these malignancies, head and neck cancer (HNC), the seventh most common cancer worldwide, remains a major contributor, accounting for approximately 940,000 new cases and 480,000 deaths annually worldwide, according to the Global Cancer Observatory estimates for 2022 [[2], [3], [4]]. Over 90% of HNCs are squamous cell carcinomas (HNSCC) arising from the mucosal linings of the oral cavity, oropharynx, larynx, or hypopharynx, with additional cases originating from cutaneous squamous cells. Less commonly, HNCs also present as adenocarcinomas of salivary or head and neck glands. These malignancies exhibit substantial biological and clinical heterogeneity driven by anatomical location and tumor origin [5].

Management of HNC is guided by tumor- and patient-related factors, with treatment typically involving surgery, radiotherapy (RT), and/or systemic therapy including chemotherapy, targeted agents, or immunotherapy, delivered as single or combined modalities depending on disease stage and location [6]. RT plays a central role in HNC management and is used in approximately 75% of patients with HNSCC, either as a primary treatment or as adjuvant therapy following surgical resection, due to its efficacy in achieving local tumor control in early-stage disease [[7], [8], [9]].

RT may be administered using photon-based intensity-modulated RT (IMRT) or proton RT (PRT) [10]. Both modalities utilize high-energy particles to induce tumor cell death through direct DNA damage and indirect generation of reactive oxygen species (ROS), which impairs cancer cell replication. Although healthy cells possess more efficient DNA repair mechanisms, high-dose radiation exposure can overwhelm these reparative pathways, which results in cumulative tissue injury [11]. Consequently, exposure to high doses of RT is associated with significant adverse effects on the soft and hard tissues of the oral cavity, influenced by the irradiated tissue volume, fractionation scheme, and treatment modality [9,12].

One such complication is osteoradionecrosis (ORN), a rare sequela of RT characterized by exposed or radiographically non-viable necrotic bone that fails to heal for at least 3-6 months. ORN may develop months to years after RT, most commonly 4 months to 2 years post-RT treatment. Radiologic features include irregular osteolytic destruction, cortical disruption, bone sequestration, and/or pathological fracture in the absence of tumor recurrence [13,14]. Numerous local and systemic risk factors have been associated with ORN development, including tumor site and stage, proximity to bone, radiation field and dose, poor oral hygiene, dental trauma or extraction before or after RT, and systemic comorbidities such as smoking, alcohol use, immunodeficiency, and infection [10,15,16].

Clinically, ORN symptoms can range from asymptomatic exposed bone to severe pain, disfiguration, pathological fracture, and functional impairment, resulting in significant reductions in quality of life [17]. At the pathophysiologic level, radiation induces endothelial injury, microvascular compromise, progressive hypoxia, hypocellularity, and fibrosis, as well as impaired bone remodeling and wound healing capacity [18]. Advances in imaging modalities, such as computed tomography (CT) and magnetic resonance imaging (MRI), have improved visualization of these underlying microenvironmental changes revealing osteolysis, cortical disruption, medullary trabecular loss, marrow signal abnormalities, and associated soft tissue pathology [14].

Over time, ORN classification systems have evolved to reflect increasing understanding of disease severity and progression, with corresponding treatment strategies ranging from conservative management (e.g., optimization of oral hygiene and antibiotic therapy) to aggressive surgical intervention in advanced stages. The first widely recognized staging system was proposed by Dr. Robert Marx in 1983 [19], grounded in the concept of radiation-induced hypoxia, hypocellularity, and hypovascularity. Subsequent classifications expanded upon this foundation, including the widely used Notani classification, which emphasizes the anatomic extent of disease, and more recent efforts such as the proposed ClinRad system, which integrates clinical findings with radiological features to provide a more comprehensive assessment of ORN severity [20]. These systems have improved clinical stratification and surgical decision-making but remain largely descriptive and retrospective, often offering limited insight into the underlying biological mechanisms that govern disease initiation, persistence, and progression.

Current treatments vary by disease severity and include optimization of oral hygiene, antibiotics, minimal surgical debridement, hyperbaric oxygen therapy (HBOT), and medical management with pentoxifylline and tocopherol, while advanced disease often necessitates aggressive surgical resection with osseous free-flap reconstruction [21]. These approaches address important downstream manifestations of ORN, including infection, vascular insufficiency, fibrosis, and structural loss, but durability is variable and biological competence of irradiated tissue is not necessarily restored. Persistent dysfunction within the vascular, stromal, immune, and bone-resident cell populations underscores the need for therapeutic strategies that address the underlying biological competence of irradiated tissue, rather than its downstream manifestations alone.

When ORN resection produces substantial mandibular defects, reconstruction must restore both structural continuity and biological healing capacity. Although autologous vascularized bone remains the reconstructive standard for advanced disease, tissue engineering has emerged to offer approaches that combine structural matrices with cells or regenerative signals. Biomaterials can mimic selected features of native bone extracellular matrix (ECM), support osteoconduction, and serve as localized delivery vehicles for growth factors, nucleic acids, extracellular vesicles (EVs), or other therapeutics [22]. In ORN, they serve not only to fill structural defects but also to modify the irradiated microenvironment in which regeneration must occur.

Ionizing radiation (IR) disrupts cellular behavior and regenerative potential, particularly in bone tissue, where it compromises healthy bone formation through mechanisms such as cell-cycle arrest, DNA damage, and radiation-induced cell death [23,24]. IR also generates excessive oxidative stress, which leads to sustained DNA damage responses that can drive long-lived changes in cell fate [25]. Increasing evidence indicates that these stress responses induce stress-induced premature senescence (SIPS) in multiple bone-resident cell populations, characterized by irreversible proliferative arrest and the emergence of the senescence-associated secretory phenotype (SASP) [[26], [27], [28], [29]]. Senescent cells persist in a hyporeplicative yet metabolically active state and are characterized in part by the expression of the cell-cycle inhibitor p16^INK4^ [30,31].

Importantly, the pathological accumulation of senescent cells fundamentally alters the tissue microenvironment through sustained SASP factor secretion, thereby causing chronic inflammation, ECM remodeling, and bystander senescence in neighboring cells [32,33]. These effects are reinforced through autocrine and paracrine feedback loops, resulting in self-propagating amplification of senescence-associated signaling [34].

In this review, we address RT-associated toxicity to the bone microenvironment, critically evaluate current classification systems and treatment strategies for ORN, and discuss emerging regenerative interventions. Rather than positioning any single pathway as sufficient to explain treatment resistance, we present ORN as a multibarrier regenerative disorder involving persistent vascular, immune, metabolic, cellular, and matrix dysfunction. We therefore further explore senotherapeutic approaches targeting radiation-induced cellular senescence as a complementary strategy to restore tissue repair capacity. These perspectives serve as evidence for the use of regenerative biomaterials and senescence-modulating therapies in the treatment of ORN.

### 1.1 Scope and novelty of this review

Prior reviews of ORN have addressed clinical classification and surgical management, radiation-induced vascular and fibrotic injury, and general bone tissue engineering strategies for craniofacial reconstruction. Separately, reviews of cellular senescence have examined its role in aging-related bone loss and the emerging pharmacology of senotherapeutic agents. However, no existing review has integrated these perspectives to frame ORN specifically as a senescence-driven regenerative failure amenable to targeted biological intervention. This review addresses that gap by synthesizing current understanding of radiation-induced microenvironmental injury across bone-resident cell populations, critically evaluating existing ORN classification and treatment strategies, and proposing senotherapeutic approaches as a complementary avenue to restore tissue repair capacity. We further examine how regenerative biomaterials, including injectable hydrogels, mineral composite scaffolds, cryogel platforms, and ceramic constructs, may serve as localized delivery vehicles for senotherapeutics, osteogenic signals, and cell-based therapies within the irradiated bone niche [[35], [36], [37], [38]].

## **2** **Radiation**-**induced injury to the bone microenvironment**

Bone (Fig. 1A) is a composite material composed of an inorganic mineral phase, hydroxyapatite (HA, Ca_10_(PO_4_)_6_(OH)_2_), integrated with an organic matrix of type I collagen, non-collagenous proteins, and lipids [39]. The bone microenvironment is maintained through tightly coordinated interactions among resident bone cells: osteoblasts, osteoclasts, and osteocytes. In addition to these cell types, mesenchymal stem cells (MSCs), immune cells, and local endothelial populations play critical roles in regulating osteogenic differentiation and bone remodeling [40]. Neural elements and bone marrow adipocytes further contribute to microenvironmental regulation through neurovascular signaling and paracrine modulation of osteogenesis, processes that are increasingly recognized as radiation sensitive [41].

**Fig. 1:**
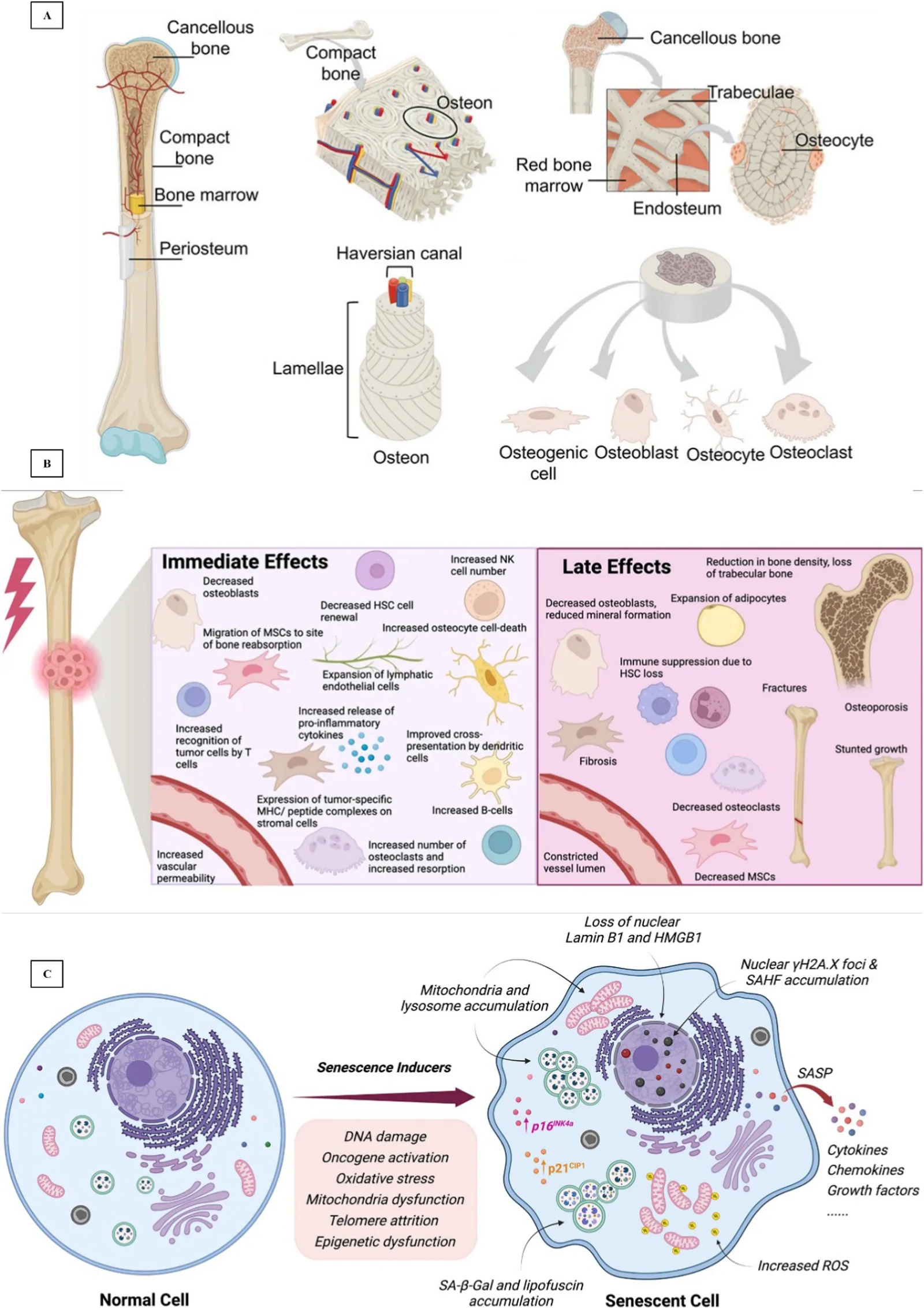
**Bone structure and cellular responses to radiation and senescence.** (A) Hierarchical organization of bone, including compact and cancellous bone, osteons, trabeculae, and major bone cell types. (B) Immediate and late effects of radiation on the bone microenvironment. (C) Major inducers and hallmarks of cellular senescence. (A) Reproduced with permission from Ref. [42]. (B) Reproduced from Ref. [43] under CC BY-NC-ND 4.0. (C) Reproduced from Ref. [44] under CC BY 4.0.

Radiation has immediate and late effects (Fig. 1B) on both the bone ECM and cellular populations within bone tissue. Vascular, cellular, and ECM injury induced by radiation drives the formation of a chronically hypoxic, hypocellular, and hypovascular bone microenvironment, ultimately predisposing tissue to ORN [45]. Radiation additionally alters ECM composition and mechanics through collagen fragmentation, increased crosslinking, and impaired matrix turnover, reducing tissue compliance and limiting cell-matrix signaling required for regeneration. The mandible is the most frequent site of ORN [46], a vulnerability often attributed to its dense mineral structure, limited blood supply, high baseline remodeling demands, and proximity to the oral microbiome [17,47]. Accordingly, ORN risk correlates more strongly with the volume of bone exposed to radiation than with peak dose alone [48].

Bone integrity and regenerative capacity depend on the coordinated homeostasis of multiple cell populations. Importantly, radiation does not affect all bone-resident cells uniformly. Differences in radiosensitivity, proliferative capacity, lifespan, metabolic activity, and niche dependence lead to distinct responses among osteoblasts, osteoclasts, osteocytes, immune cells, MSCs, endothelial populations, and the native microbiome, disrupting patterns of bone repair and homeostasis within the bone microenvironment [49]. These heterogeneous responses uncouple normally coordinated osteogenic, angiogenic, and immune processes, producing a microenvironment characterized by progressive vascular loss, impaired remodeling, aberrant inflammatory signaling, and reduced regenerative plasticity. These changes underlie the transition from acute radiation injury to the chronic, self-sustaining pathology characteristic of ORN. The following sections detail the radiation responses of individual bone-resident cell populations that underlie ORN pathogenesis.

### **2.1** **Cell**-**specific radiation responses relevant to ORN pathogenesis**

#### 2.1.1 Osteoblasts and osteoprogenitors

Osteoblasts are derived from MSCs and are responsible for bone formation through the deposition of osteoid, including type I collagen and other ECM proteins. During bone growth and remodeling, osteoblasts have a relatively short lifespan of approximately 100 days, during which they proliferate, synthesize matrix, and subsequently undergo one of three fates: apoptosis, terminal differentiation into osteocytes embedded within the bone matrix, or transition into quiescent bone-lining cells on the bone surface [50].

Damage to osteoblasts and their progenitors is a primary contributor to reduced bone mineral density following irradiation [[51], [52], [53]]. Radiation reduces osteoblast number and matrix formation, and both *in vitro* and *in vivo* studies demonstrate impaired osteoblast proliferation and differentiation, cell-cycle arrest, decreased collagen production, and increased sensitivity to apoptotic stimuli [51,54]. Because of their short lifespan, continual repopulation of osteoblasts through osteogenic differentiation of MSCs is essential. However, in irradiated tissue, MSC numbers, clonogenicity, and osteogenic differentiation capacity are reduced, delaying recovery of functional osteoblast populations [55]. Radiation also promotes a shift of MSC fate toward adipogenesis at the expense of osteogenesis, contributing to marrow adiposity and further suppression of bone formation. Oxidative stress contributes significantly to early osteoprogenitor damage and persistent lineage dysfunction [56].

#### 2.1.2 Osteocytes

The effects of radiation on osteocyte viability and function are heterogeneous and context-dependent. Some studies report osteocyte loss following high-dose irradiation, particularly within cortical bone, resulting in empty lacunae [55]. In contrast, other models demonstrate relative osteocyte survival, albeit with persistent functional impairment [57]. Importantly, radiation alters osteocyte signaling even in surviving cells. Irradiated osteocytes exhibit disrupted dendritic architecture, persistent γH2AX DNA damage foci, and dysregulation of key regulators of bone remodeling, including increased sclerostin and altered RANKL/OPG signaling. These changes impair mechanotransduction and osteocyte-mediated coordination of bone remodeling. When co-cultured with osteoclast precursor cells, irradiated osteocytes significantly promote osteoclastogenesis, implicating osteocytes as active drivers of radiation-induced bone resorption rather than passive victims of injury [58].

#### 2.1.3 Osteoclasts

Irradiation induces an early increase in osteoclast number and activity, likely contributing to rapid bone loss during the early stages of ORN. Serum markers of osteoclast activity, including tartrate-resistant acid phosphatase 5b (TRAP5b), rise within 24 h of irradiation, with altered osteoclast morphology and increased resorptive activity observed within days in preclinical models [59]. This early resorptive response is thought to be driven by inflammatory and osteocyte-derived cues rather than intrinsic osteoclast radioresistance. Notably, pre-osteoclasts, but not fully differentiated osteoclasts, efficiently clear apoptotic cells through efferocytosis (phagocytic removal of apoptotic debris) [60]. In the irradiated niche, impaired efferocytosis may contribute to the accumulation of apoptotic debris and sustained inflammatory signaling that perpetuates the chronic, self-sustaining pathology characteristic of established ORN.

The majority of early bone loss occurs during a period when osteoblast number and bone formation remain relatively unchanged [59]. At later time points, both osteoblast and osteoclast activity decline, resulting in prolonged suppression of bone remodeling, deterioration of microarchitecture, and impaired material properties of bone tissue—hallmarks of established ORN [55].

#### 2.1.4 Hematopoietic environment

Cells within the common myeloid cell lineage give rise to monocytes, macrophages, dendritic cells, and osteoclasts. In adult mice exposed to a single 2-gray (Gy) dose of radiation, increases in myeloid and monocyte-macrophage lineage cells have been observed, accompanied by elevated expression of TRAP5b [61].

The osteoimmune environment plays an essential role in maintaining bone homeostasis, with both innate and adaptive immune cells contributing to bone regulation [47]. Due to its proximity to the oral cavity and resident oral microbiota, the mandibular bone marrow exhibits a particularly active immune environment [62].

Single-cell mapping of the alveolar bone marrow has revealed extensive crosstalk between MSCs and monocyte/macrophage populations, with macrophages representing the most interactive immune cell type with MSCs in this niche [62]. Radiation exposure disrupts this environment, driving maladaptive macrophage activation and leading to unresolved inflammation [63]. IR also drives dendritic cell maturation and inflammatory NF-κB signaling, which results in impaired BMSC migration, proliferation, and osteogenic differentiation [64].

#### 2.1.5 Oral microbiome and local inflammatory context

The oral microbiota contains over 700 microbial species, primarily consisting of *Actinobacteria, Bacteroidetes, Firmicutes, Ascomycota, Spirochaetes, and Saccharomycetes* which play a direct role in maintaining oral health and defending against external pathogens [65]. After RT, the microbial population shifts and pathogenic bacterial families prevail [66]. This shift to the bacterial families *Prevotellaceae, Fusobacteriaceae, Porphyromonadaceae, Actinomycetaceae,* and *Staphylococcaceae* ultimately alters the microbiomic balance, disrupts the immune system response, and promotes inflammation, disrupting the healthy bone remodeling processes.

#### 2.1.6 Endothelial cells & vasculature

Endothelial cells form the vascular network within bone and are critical for tissue oxygenation, metabolism, immune response, and progenitor cell trafficking [67]. Radiation disrupts endothelial integrity and vascular supply, negatively impacting nutrient delivery and promoting immune cell recruitment [68,69]. Endothelial cells also contribute to bone homeostasis through angiogenic-osteogenic coupling. Specialized type H endothelial cells promote osteogenesis by supporting osteoprogenitor expansion, while pre-osteoclasts and other myeloid cells reciprocally enhance endothelial growth through angiogenic signaling [70]. Endothelial cells also facilitate delivery of osteoblastic precursors and trophic growth factors. Radiation-induced loss of these specialized endothelial subtypes disrupts this coupling, further impairing coordinated bone repair.

In a study examining the effects of total body irradiation on bone marrow vasculature, Himburg et al. exposed C57BL/6 mice to 5- or 7.5-Gy of total body irradiation and observed acute disruption of bone marrow sinusoidal vasculature within 6-24 h at both doses. At 5-Gy, evidence of vascular recovery and vessel formation was apparent by day 14. In contrast, 7.5-Gy irradiation resulted in persistent endothelial apoptosis and delayed vascular regeneration [71]. Consistent with these findings in animal models, retrospective analysis of human mandibular bone biopsies from irradiated patients demonstrated dose-dependent reductions in vascular density and vessel area fraction compared with non-irradiated controls. Radiation doses exceeding 50-Gy preferentially reduced small-caliber vessels and inhibited neovascularization, underscoring the vulnerability of the mandibular microvasculature to radiation injury [72].

### 2.2 Persistent cellular stress states in irradiated bone

Radiation injury is not limited to the depletion of individual cell populations. Cells that survive irradiation may exhibit persistent metabolic, transcriptional, and functional abnormalities that compromise their capacity to participate in tissue repair. Among these responses, sustained oxidative stress, altered iron metabolism, mitochondrial dysfunction, and cellular senescence provide potential mechanisms linking the initial radiation damage to prolonged regenerative failure [24,73,74].

#### 2.2.1 Oxidative stress, iron dysregulation, and emerging ferroptotic mechanisms

IR generates ROS directly through radiolysis and indirectly through persistent mitochondrial dysfunction, producing oxidative damage that can extend beyond the initial exposure. Within bone, prolonged redox imbalance can impair osteogenic differentiation, alter osteoclast activity, promote inflammatory signaling, and compromise the survival and function of regenerative cell populations [75,76]. Emerging evidence suggests that disruption of cellular iron homeostasis may further amplify this oxidative environment and lead to ferroptosis [77]. Ferroptosis is an iron-dependent form of regulated cell death driven by excessive lipid peroxidation. IR can promote this form of cell death by generating ROS, promoting polyunsaturated fatty acid (PUFA) metabolism, and impairing the antioxidant GPX4 pathway [78]. IR both breaks down ferritin to release iron and induces lysis of red blood cells following radiation exposure [79]. This increase in iron accumulation in the bone marrow has been shown to induce ferroptosis in granulocyte-macrophage progenitor cells [80]. Additionally, iron accumulation may disrupt the bone environment and impair osteoblast activity, promote osteoclastogenesis, and polarize macrophages toward the inflammatory M1 phenotype [81]. The contribution of radiation-induced iron dysregulation and ferroptosis to ORN pathogenesis remains unclear, and whether targeting these mechanisms offers therapeutic benefit warrants direct investigation in clinically relevant mandibular models.

### 2.3 Senescence-driven failure of bone healing in ORN

Across bone-resident cell populations, radiation-induced senescence (Fig. 1C) produces convergent defects in regeneration, immune resolution, and angiogenic-osteogenic coupling. Table 1 synthesizes these cell-specific senescence-associated phenotypes and their contributions to ORN pathogenesis.

**Table 1:** Radiation effects on cell populations within the bone microenvironment.

Cell Type	Normal Function	Radiation/Senescence-Associated Phenotype	Pathogenic Contribution to Bone/ORN	References
Osteoblasts	Synthesize bone matrix; regulate mineralization; provide anabolic signals for bone formation	Decreased osteogenic differentiation and mineralization; increased apoptosis and/or senescence; persistent cell-cycle arrest; SASP secretion including pro-inflammatory cytokines (e.g., IL-6) and matrix remodeling enzymes (e.g., MMPs)	Suppressed bone formation and impaired repair; establishment of a pro-inflammatory/-fibrotic niche that limits regeneration and osteoblast repopulation	[[54], [55], [56],^58^,^59^]
Osteocytes	Mechanosensors; coordinate osteoblast-osteoclast coupling via SOST and RANKL/OPG signaling	Persistent DNA damage signaling; increased SOST expression; impaired lacunar-canalicular network/function; SASP-promoting osteoclastogenesis	Defective remodeling and increased fragility; suppression of osteoblast activity and promotion of osteoclast-mediated resorption; contribution to chronic bone loss and impaired healing	[58,82]
Osteoclasts	Resorb bone in regulated balance with osteoblast-mediated formation	Early radiation-induced increase in osteoclast number and activity; uncoupled resorption driven by inflammatory and osteocyte-derived cues; altered differentiation dynamics	Acute bone loss and microarchitectural deterioration; predisposing irradiated bone to failure of remodeling and necrosis	[59,60]
Bone marrow MSCs (BMSCs)	Progenitors for osteoblasts, chondrocytes, adipocytes; support stromal niche and regenerative signaling	Radiation-induced BMSC senescence and paracrine dysfunction; reduced clonogenicity and osteogenic potential; shifting toward adipogenic programs and impaired regenerative signaling	Depletion of osteoprogenitor pool; impaired regeneration; increased marrow adiposity; delayed/failed healing in irradiated bone	[51,54,56,82,83]
Myeloid cells (macrophages/monocytes/myeloid-derived suppressor cells [MDSCs])	Regulate inflammation; coordinate tissue repair; support osteogenesis and angiogenesis	Senescence-linked immune dysfunction; persistent pro-inflammatory (M1) polarization; SASP-like secretion of inflammatory and pro-fibrotic mediators; contributes to chronic inflammation and fibrosis	Persistent inflammation and fibrosis; defective immune-mediated repair; pro-fibrotic signaling that degrades tissue quality and healing capacity	[60,[84], [85], [86]]
Endothelial cells/bone vasculature	Maintain vascular integrity and permeability; regulate oxygen and nutrient delivery; support osteogenesis-angiogenesis coupling	Endothelial activation/dysfunction after irradiation; oxidative stress and inflammatory signaling; increased permeability and vascular injury; impaired angiogenic potential	Hypovascular/hypoxic microenvironment; impaired nutrient delivery and progenitor trafficking; defective repair and chronic injury signaling	[[68], [69], [70]]

## 3 Classification and current treatment approaches for ORN

ORN is a late complication of RT with highly variable clinical presentation, progression, and severity, reflecting substantial heterogeneity in disease course and treatment response. The onset of ORN can vary but most commonly occurs between 4 months and 2 years following RT [45]. Radiologic features include irregular osteolytic destruction, cortical disruption, bone sequestration, and/or pathological fracture in the absence of tumor recurrence [13,14].

Clinically, ORN displays marked heterogeneity in presentation, progression, and severity, reflecting substantial variability in disease course and treatment response [45,87]. Despite decades of clinical experience, ORN management is largely guided by retrospective studies, expert opinion, and institutional practice patterns, as high-quality randomized controlled trials are scarce [[88], [89], [90]]. As a result, treatment strategies are highly variable and largely empiric, reflecting the evolving, and at times overlapping, pathophysiological ORN models that have shaped therapeutic approaches over time [[91], [92], [93]].

### 3.1 ORN classification systems

Several classification systems for ORN have been proposed over the years, reflecting evolving concepts of disease severity, anatomical involvement, and clinical progression. Among these, the classification developed by Notani et al. is widely used due to its simplicity and reliance on the vertical extent of mandibular involvement relative to the inferior dental canal [94].

In this system, Stage I ORN is confined to the dentoalveolar bone, with preservation of the mandibular basal bone and structural continuity [95]; these lesions are biologically compromised but mechanically stable. Stage II ORN extends into the mandible above the inferior dental canal, indicating more extensive necrosis while maintaining continuity. This stage often represents a transitional disease state in which regenerative or conservative strategies may still be feasible depending on vascular status and disease chronicity. Accordingly, conservative treatment strategies are typically employed in symptom-free or minimally symptomatic patients with early to moderate ORN (Notani stages I-II). When conservative strategies fail or disease progresses despite treatment, advancement to Stage III generally signals the need for surgical intervention [96]. Stage III ORN involves necrosis below the inferior dental canal and/or is associated with pathological fracture or cutaneous fistula, reflecting advanced structural and vascular compromise with loss of mandibular stability [87,96]. This stage most commonly presents as a critical-size bone defect and typically requires combined reconstructive and regenerative approaches rather than biological modulation alone [97,98].

### 3.2 Historical evolution of ORN pathophysiological models

Early descriptions of ORN emphasized the role of bacterial infection, trauma, and radiation-induced tissue injury. Watson and Scarborough subsequently observed bacterial colonization in affected tissues and described associated structural changes, including osteoblast and osteocyte loss, arterial wall thickening, and inflammatory infiltration of bony cavities; these observations culminated in the early characterization of ORN as a consequence of radiation, trauma, and infection [99].

Meyer formalized the radiation-trauma-infection theory in 1970, classifying ORN as a form of osteomyelitis initiated by mucosal trauma [100]. This framework dominated clinical practice for over a decade and established antibiotics combined with surgical debridement as the standard treatment approach. However, this model was challenged by Daly and Drane, and later Marx, after reports that around 40% of ORN developed without identifiable trauma [101,102].

### 3.3 Hypoxia-based and fibroatrophic models

The limitations of the infection-based paradigm prompted Marx to propose the hypoxic-hypocellular-hypovascular theory, postulating that radiation induces progressive vascular injury and fibrosis, leading to hypoxic, hypocellular, and hypovascular tissue incapable of sustaining normal remodeling or wound healing [101]. This theory directly motivated the clinical adoption of HBOT for both the prevention and treatment of ORN [45].

Building upon Marx's framework, Delanian and colleagues later advanced the radiation-induced fibroatrophic (RIF) model, which reframed ORN as a dynamic and progressive process characterized by chronic inflammation, endothelial dysfunction, excessive fibrosis, and tissue atrophy rather than a static hypoxic state [103]. This framework shifted attention toward progressive vascular and fibrotic remodeling, and informed antifibrotic strategies such as pentoxifylline and tocopherol [104,105]. Importantly, this shift emphasized radiation-induced endothelial injury as an early and sustained driver of disease, linking vascular dysfunction to downstream hypoxia, inflammation, and fibrotic remodeling [59,106].

### 3.4 Current treatment strategies

Treatment strategies for ORN are stratified by disease severity and progression. Preventative management remains critical and includes pre-RT dental evaluation, optimization of oral hygiene, atraumatic dental care, and careful radiation planning [107]. Consideration of patient-specific risk factors, such as diabetes, smoking, dental extractions, radiation dose/technique, and concurrent chemotherapy, is essential in reducing ORN incidence [93]. Current clinical management strategies for ORN, their proposed mechanisms of action, and typical indications across disease severity are summarized in Table 2.

**Table 2:** Current treatment modalities for ORN, corresponding mechanisms, and clinical indications.

Treatment Modality	Primary Mechanism of Action	Typical Indication	References
Conservative care (oral hygiene, local irrigation, systemic antibiotics)	Reduces bacterial load; controls secondary infection; removes superficial necrotic debris	Early or mild ORN	[93,107,108]
Local surgical debridement/sequestrectomy	Removes devitalized bone while preserving mandibular continuity	Early to moderate ORN	[89,107]
Hyperbaric oxygen therapy (HBOT)	Increases tissue oxygen tension; promotes angiogenesis; exerts bacteriostatic effect	Early to moderate ORN (≤65-Gy); adjunctive use in severe ORN	[[109], [110], [111], [112], [113]]
Pentoxifylline + Tocopherol (PENTO)	Improves microvascular perfusion; reduces oxidative stress; partial inhibition of transforming growth factor-β1 (TGF-β1)-mediated fibrotic signaling	Early to moderate ORN	[104,106,114]
PENTO + Clodronate (PENTOCLO)	Adds antiresorptive inhibition of osteoclast-mediated bone loss to vascular and antifibrotic modulation	Persistent or refractory ORN after PENTO	[89,104,115]
Surgical resection with microvascular free-flap reconstruction	Removes necrotic bone; restores mandibular continuity and function	Advanced ORN	[116,117]
Therapeutic ultrasound/low-level laser therapy (LLLT)	Mechanical or photobiomodulation to enhance perfusion, angiogenesis, and tissue remodeling	Adjunct to surgery or PENTO-based therapy	[[118], [119], [120]]

Early and moderate-stage ORN have been traditionally managed through conservative approaches aimed at minimizing infection, promoting wound healing, and limiting disease progression. These include oral hygiene optimization, oral or parenteral antibiotics, and local surgical interventions such as sequestrectomy or saucerization [89,107,108].

#### 3.4.1 Hyperbaric oxygen therapy (HBOT)

HBOT emerged as a dominant early treatment modality based on the hypothesis that radiation induces hypoxia, reduced vascularity, and impaired healing [112]. HBOT involves breathing 100% oxygen under increased atmospheric pressure, increasing tissue oxygen delivery and promoting angiogenesis, collagen synthesis, and wound healing through hyperoxic tissue conditioning [121]. However, multiple studies have demonstrated that HBOT is largely ineffective as a standalone therapy and shows greater benefit only when combined with surgical debridement or resection [111,112]. As a result, its role remains controversial; some authors recommend HBOT as an adjunctive early intervention rather than definitive therapy [45].

More localized approaches have recently been investigated to increase oxygen availability within ORN lesions distinct from HBOT. Duvaud et al. reported the first intraoral use of H-MOD, an oxygenating dressing containing the extracellular hemoglobin Hemarina-M101, in two patients with advanced mandibular ORN [122]. Hemarina-M101 acts as a local oxygen carrier, providing oxygen within the wound environment without requiring increased atmospheric pressure. One patient demonstrated initial improvement in mucosal coverage followed by subsequent disease progression, whereas the second achieved complete mucosal healing without exposed bone at six months. The mixed clinical responses to both systemic hyperbaric oxygen and localized oxygenation suggest that tissue hypoxia alone cannot fully account for the persistence and progression of ORN, pointing toward a more complex, multifactorial pathogenesis of ORN [113].

#### 3.4.2 Surgical management

In patients who fail conservative therapy, surgical management is employed to manage symptoms, restore function, and improve overall quality of life. Surgical options include debridement, sequestrectomy, local tissue rearrangement, marginal mandibulectomy, and, in cases of advanced disease, microvascular free-flap reconstruction [116]. In persistent ORN, mandibular resection is often necessary to prevent facial collapse, deformity, and severe functional disability. In these cases, the necrotic bone is removed and vascular bone grafts, most commonly fibular free flaps, are used to restore mandibular continuity and function [117]. Free-flap reconstruction demonstrates high success rates, with healing and disease control reported in over 90% of patients across multiple studies [[123], [124], [125], [126], [127]]. However, free-flap reconstruction carries significant morbidity, including donor-site complications, prolonged operative time, and the requirement for microsurgical expertise. Furthermore, even successful reconstruction does not address the underlying biological dysfunction of the irradiated microenvironment, leaving residual tissue susceptible to ongoing degeneration.

Emerging comparative data increasingly support surgical intervention as an effective strategy even in earlier-stage disease. In a prospective clinical study, patients treated surgically with sequestrectomy, resection, or free flap reconstruction demonstrated higher rates of pain relief (90% vs. 70%) and complete disease resolution (83% vs. 50%) compared with those managed conservatively using antibiotics, HBOT, or pentoxifylline-tocopherol regimens [128].

#### 3.4.3 Pharmacologic and adjunctive therapies

While surgical approaches address structural and symptomatic manifestations of disease, pharmacological therapies aim to target radiation-induced fibrosis and other underlying pathophysiologic processes. Importantly, many conservative and adjunctive therapies exhibit delayed, partial, or transient clinical responses, with relapse or disease progression commonly reported during extended follow-up [113]. Pentoxifylline-based regimens represent one of the most clinically substantiated pharmacologic strategies for ORN. Pentoxifylline enhances microvascular perfusion through hemorheological and anti-TNF-α effects, while tocopherol (vitamin E) provides antioxidant activity and partially attenuates TGF-β1-mediated fibrotic signaling [90]. Together, the pentoxifylline-tocopherol (PENTO) regime targets key vascular, inflammatory, and fibrotic pathways implicated in radiation-damaged bone. A systematic review and meta-analysis encompassing over 200 patients demonstrated that PENTO therapy resulted in clinical improvement or complete resolution in a majority of cases, particularly in early to moderate disease stages, often reducing the need for surgical intervention [114]. In patients with persistent or refractory disease, the addition of clodronate (PENTOCLO) further suppressed osteoclast-mediated bone resorption and has shown efficacy in a Phase II trial of 54 patients, with universal clinical improvement and a median time to complete recovery of approximately nine months [115].

Other adjunctive modalities aim to improve conventional treatments and ORN outcomes. Low-intensity therapeutic ultrasound and low-level laser therapy (LLLT) have been explored as adjuncts to improve perfusion and angiogenesis through mechanical or photobiomodulatory mechanisms. Ultrasound exerts mechanical stimulation that can activate pro-healing biochemical signaling pathways. In a canine model, ultrasound treatment was not sufficient as a standalone therapy but was associated with measurable improvements in bone healing and remodeling within irradiated tissue [118]. LLLT enhances mitochondrial ATP production and modulates redox-sensitive signaling pathways, activating transcription factors involved in angiogenesis, ECM deposition, and inflammation, and has been used as a supplemental therapy alongside conservative management [119,120].

#### 3.4.4 Persistent cellular dysfunction and emerging gaps

Despite these advances, none of the prevailing models fully account for persistent radiation-induced dysfunction within resident bone and stromal cell populations, including osteocytes, mesenchymal stromal cells, immune cells, and endothelial cells [111,113]. Chronic cellular stress, impaired mechanosensing, altered paracrine signaling, and SASP may represent underappreciated contributors to treatment resistance and disease chronicity [13,49]. This unresolved mechanistic gap likely underlies the limited durability of current therapies and highlights the need for strategies that directly target persistent cellular dysfunction rather than downstream fibrosis or hypoxia alone.

## 4 Emerging regenerative approaches in ORN

### 4.1 Cell-based and cell-directed regenerative strategies

Stem cells are undifferentiated cells characterized by their capacity for self-renewal and differentiation, and are broadly classified into embryonic stem cells (ESCs) and adult stem cells (ASCs) [129]. Advances in cellular reprogramming have also enabled the generation of induced pluripotent stem cells (iPSCs), which display ESC-like properties while circumventing some ethical concerns [130,131].

Among ASC populations, MSCs have gained the most attention in treatment strategies for radiation injury and ORN because of their osteogenic, angiogenic, and immunomodulatory properties. The effectiveness of these regenerative therapies depends not only on the selected cell population, but also on the timing of administration, delivery route, accompanying biomaterial (if any), and presence of osteogenic or angiogenic signals. The longevity and persistence of transplanted cells also remain poorly understood [132]. Phulpin et al. evaluated the feasibility of short-term retention of autologous BMSCs in a rat model of hindlimb irradiation [133]. Three months after a 30-Gy dose of IR to the left hindlimb, 2 × 10^6^ radiolabeled BMSCs were injected through a cortical drill hole directly into the medullary canal of the left tibia. The local transplantation resulted in 40% cell retention at one week and produced a significant enhancement in both bone blood flow and osteoblastic metabolism during the first post-treatment week. However, these therapeutic effects were temporary and diminished by two months post-injection. Systemic administration of MSCs is limited by lower rates of engraftment and can lead to unpredictable biodistribution, often sequestering cells in the lungs [134]. Additional pre-clinical and early clinical studies have explored MSC-based treatment in ORN. However, the same irradiated microenvironment that provides the rationale for MSC therapy can also limit cell survival, retention, and function. Therefore, many of these approaches incorporate cells within a biomaterial carrier to improve local retention and regenerative support. Because the contribution of these cells cannot be separated from the accompanying matrix, these combination strategies are discussed below within Section 4.2 of the biomaterial-assisted therapy section.

Research now suggests that much of the therapeutic activity of MSCs is mediated through paracrine and intercellular signaling rather than direct incorporation of cells into newly formed tissue. As a result, MSC therapies have shifted attention from MSC engraftment to strategies that reproduce MSC effects without the use of intact cells. Proposed mechanisms include: i) secretion of soluble trophic factors, including vascular endothelial growth factor (VEGF), fibroblast growth factors (FGFs), and other cytokines that support angiogenesis, cell survival, and tissue regeneration; ii) intercellular communication through cell-surface proteins, chemokines, and EVs; iii) immunomodulatory signaling through mediators such as TGF-β and interleukin-10 (IL-10); and iv) regulation of cellular stress responses as shown in Fig. 2 [135]. The discovery of these mechanisms has led to the development of MSC-derived, cell-free therapies that encourage regeneration without implantation of intact cells.

**Fig. 2:**
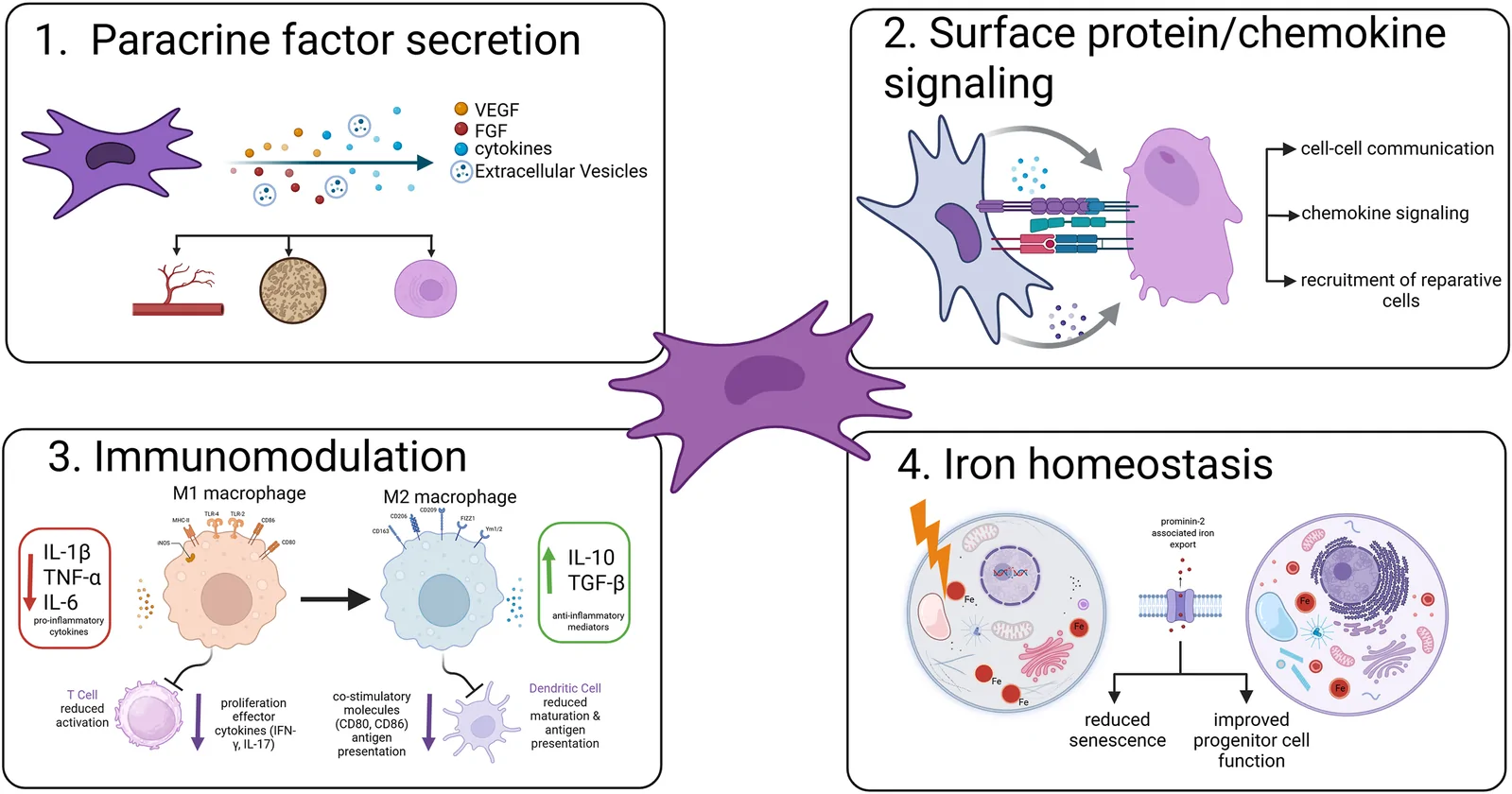
**Mechanism of MSC-based therapy in ORN.** MSCs may support regeneration within the irradiated bone microenvironment through (1) paracrine secretion of angiogenic and osteogenic factors such as VEGF, FGF, cytokines, and extracellular vesicles; (2) cell-surface protein and chemokine-mediated signaling that promotes intercellular communication, cell migration, and recruitment of reparative populations; (3) immunomodulation that shifts macrophages from a pro-inflammatory M1 phenotype (reduced IL-1β, TNF-α, and IL-6) toward an M2 phenotype (increased IL-10 and TGF-β), reduces T cell activation and effector cytokine production, and limits dendritic cell maturation and antigen presentation, thereby attenuating persistent inflammatory signaling; and (4) protection against radiation-associated cellular stress, including regulation of oxidative stress and iron homeostasis. Created in BioRender. Hixon, K. (2026) https://BioRender.com/p0lgo8r.

Stem-cell-derived EVs have begun to be investigated as a therapeutic in ORN. EVs are lipid-bound vesicles released by cells that transport proteins, lipids, messenger RNAs, microRNAs, and other regulatory molecules between cells [136]. MSC-derived vesicles can reproduce many of the angiogenic, immunomodulatory, and anti-apoptotic effects associated with the MSC secretome while avoiding some of the challenges related to cell survival and uncontrolled differentiation [137,138]. Ohara et al. evaluated EVs derived from human dental pulp stem cells in irradiated rat bone marrow cells and in a rat model of jaw ORN [139]. EV treatment reduced the expression of senescence-associated genes in irradiated cells *in vitro* (Fig. 3Bi), which suggested that the vesicles partially suppressed or modified the radiation-induced senescent phenotype. *In vivo*, intravenous administration increased new bone formation and improved gingival coverage of the irradiated jaw (Fig. 3Bii-iii). Rather than replacing damaged cells directly, the EVs appeared to modify the behavior of endogenous irradiated cells.

**Fig. 3:**
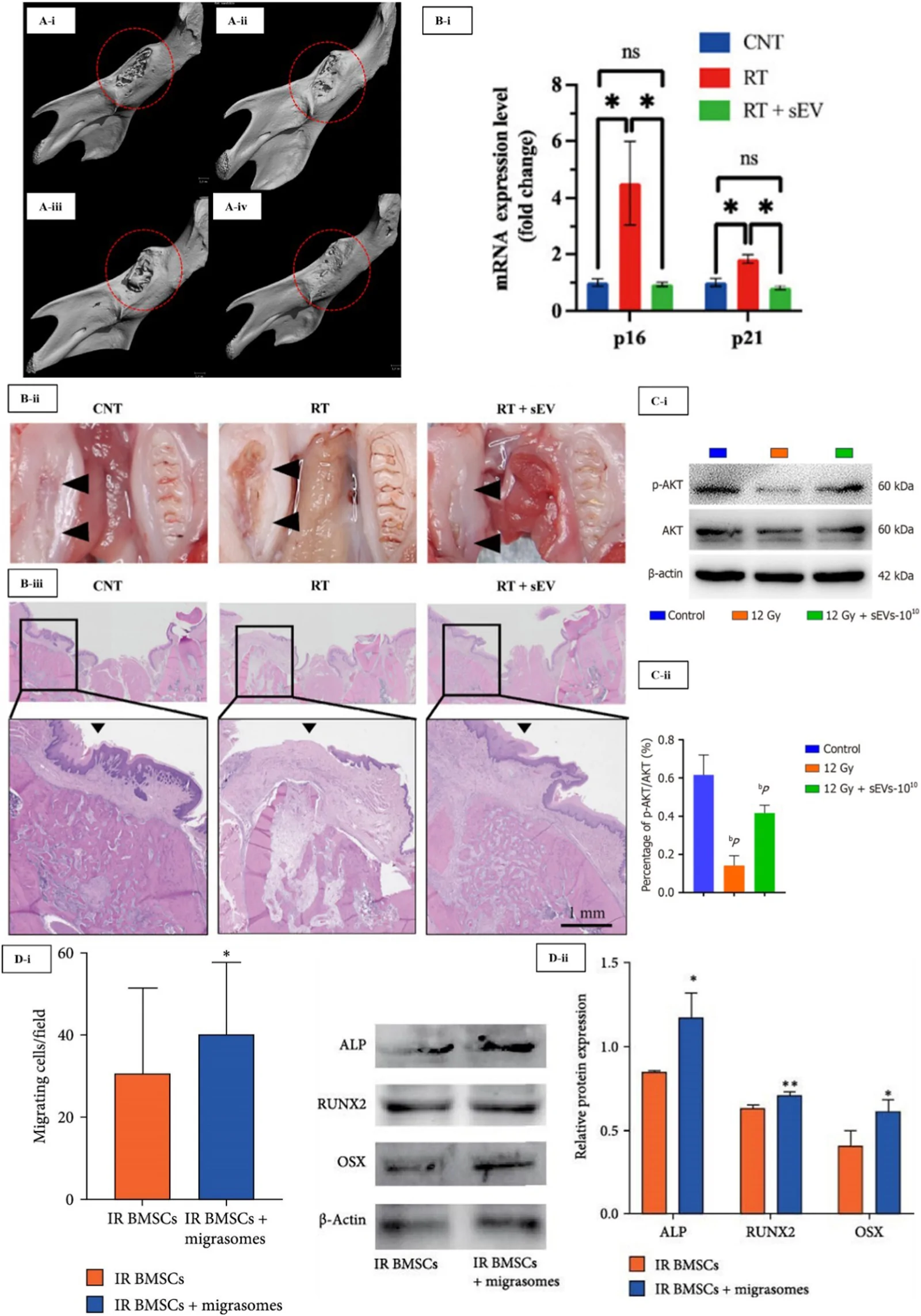
**Stem cell- and EV-focused approaches for ORN.** (A) Three-dimensional (3D) microCT images of irradiated rat extraction sockets: (i) Matrigel-4, (ii) tonsil-derived MSC-4, (iii) Matrigel-1, and (iv) tonsil-derived MSC-1 groups. Complete bone regeneration was observed in the tonsil-derived MSC-1 group. (B) Effects of human dental pulp stem cell-derived small EVs in a rat model: (i) RT-PCR analysis of senescence-associated mRNA expression; (ii) representative mandibular specimens, with black arrowheads indicating extraction sites; and (iii) representative hematoxylin and eosin (H&E)-stained mandibular sections, with black arrowheads indicating enlarged extraction sites. Scale bar: 1 mm. (C) (i) Western blot and (ii) quantitative analyses of AKT and phosphorylated AKT expression in BMSCs under control, 12-Gy irradiation, and 12-Gy irradiation plus human amniotic MSC-derived small EV treatment. (D) Effects of CYR61-associated migrasomes on irradiated BMSCs: (i) Transwell migration quantification and western blots of ALP, RUNX2, and OSX; (ii) quantification of ALP, RUNX2, and OSX protein expression. (A) Reproduced with permission from Ref. [140], John Wiley & Sons. (B) Reproduced from Ref. [139] under CC BY-NC-ND 4.0. (C) Adapted from Ref. [141] under CC BY-NC 4.0; changes include selection and relabeling of panels. (D) Reproduced from Ref. [142] under CC BY 4.0.

A related study investigated small EVs derived from human amniotic MSCs in a rat model of jaw ORN, and in irradiated BMSC and endothelial-cell cultures [141]. Local EV administration improved bone formation and vascular regeneration within the irradiated jaw. The vesicles also restored aspects of osteogenic function in irradiated BMSCs and supported endothelial cell activity. Mechanistic analysis implicated activation of the phosphoinositide 3-kinase/protein kinase B pathway (PI3K/AKT) signaling (Fig. 3Ci-ii). This pathway regulates cell survival, metabolism, proliferation, migration, and differentiation, and is frequently suppressed or dysregulated following cellular stress [143]. Activation of PI3K/AKT signaling may therefore help irradiated progenitor cells resist apoptosis, recover osteogenic activity, and interact more effectively with regenerating vascular cells [144]. The ability of EVs to simultaneously support osteogenesis and angiogenesis is important because successful bone regeneration requires coordinated development of mineralized tissue and a functional blood supply [145].

Additional approaches aim to enhance EV activity to specifically target the irradiated environment by modifying parent cells before isolation of the EVs. Zhang et al. preconditioned BMSCs with cerium oxide nanoparticles (CeONP) and subsequently isolated the resulting exosomes for treatment of radiation-induced jaw bone injury [146]. By cycling between Ce^3+^ and Ce^4+^ oxidation, CeONP-conditioned exosomes reduced radiation-associated ROS accumulation, restored osteogenic differentiation in irradiated BMSCs, and improved bone regeneration in an *in vivo* irradiated jaw.

Notably, MSC-mediated therapeutics extend beyond EVs. Yan et al. examined strategies for recovering the regenerative activity of irradiated BMSCs utilizing migrasomes derived from healthy BMSCs [142]. Migrasomes are organelles formed along retraction fibers during active cell movement, serving as specialized transport vehicles that deposit bioactive proteins and RNAs into the microenvironment. In this study, a 2-Gy radiation dose impaired BMSC migration and osteogenic differentiation without substantially reducing cell viability, showing that cells can remain alive after irradiation while losing important regenerative functions. The ECM-associated protein cysteine-rich angiogenic inducer 61 (CYR61) was transported through migrasomes and promoted the recovery of BMSC migration and osteoblastic differentiation (Fig. 3Di-ii). CYR61-containing migrasomes activated integrin αvβ3 and extracellular signal-regulated kinase signaling, which connected ECM recognition with intracellular pathways involved in migration and osteogenesis. These findings further demonstrate that radiation injury does not simply reduce the number of available progenitor cells, but can produce persistent functional abnormalities in the surviving cell population [147].

Beyond transplantation of cells or their derivatives, research has investigated physical or molecular interventions to modify radiation-induced dysfunction without requiring exogenous cellular engraftment or structural scaffold implantation. Akintoye et al. evaluated an orally delivered, plant-bioencapsulated CTB-Pro-IGF-1 fusion protein in a rat model of mandibular radiation injury [148]. IGF-1 is an osteoanabolic growth factor that supports progenitor-cell activity and bone formation. Oral CTB-Pro-IGF-1 treatment for two weeks significantly improved regeneration of the irradiated jaw, with microCT demonstrating increases in bone volume, mineral density, trabecular thickness, and trabecular number. Histological analysis similarly demonstrated greater viable bone and osteocyte preservation with reduced fibrous healing [148].

Zhang et al. found that irradiation disrupted cytoskeletal organization and impaired cellular viability, migration, and osteogenic differentiation in rat mandibular MSCs [149]. The RhoA/ROCK pathway regulates actin cytoskeletal organization, cell adhesion, contractility, migration, and mechanotransduction [150]. When they tested treatment with low-intensity pulsed ultrasound (LIPUS), they found these functions were partially restored through modulation of RhoA/Rho-associated protein kinase signaling. Recovery of this pathway may restore endogenous MSCs’ ability to migrate into damaged tissue, respond to the surrounding matrix, and undergo osteogenic differentiation.

Multiple, independent strategies have been explored to confront radiation-induced damage of the bone microenvironment, with promising results. However, advanced ORN often involves substantial structural loss in addition to disruption of the microenvironment. Biomaterial-based solutions aim to not only address the underlying structural support of regeneration, but also improve therapy retention and localization. Biomaterial-based strategies can act as both a scaffolding platform as well as a localized delivery system for cells, EVs, growth factors, and other bioactive therapeutics.

### 4.2 Biomaterial- and scaffold-based regenerative strategies

A wide range of bone grafting and reconstructive techniques have been used to restore mandibular defects [[151], [152], [153], [154]]; however, many of these approaches are limited by donor-site morbidity, high cost, and the low availability of the autogenous graft material, which remains the clinical gold standard [155,156]. These limitations are further compounded in ORN, where radiation-induced hypovascularity, fibrosis, and cellular dysfunction weaken graft integration and healing [90]. Consequently, there has been sustained interest in the development of tissue-engineered alternatives that employ synthetic and biologically active materials to promote bone regeneration in irradiated tissue [157]. In this context, natural and synthetic composite scaffolds, ceramic biomaterials, osteogenic and angiogenic growth factors, and stem cell therapies are increasingly being integrated into combination strategies to support the healing of irradiated bone tissue (Table 3) [135,35,36,158,159]. Thus, as the field of tissue engineering evolves, inert bone substitute materials that have been historically used as passive space holders during the healing process are now insufficient on their own to drive true regeneration [160].

**Table 3:** Comparison of biomaterial classes for senotherapeutic delivery in irradiated bone.

Biomaterial Class	Composition	Degradation	Mechanical Properties	Drug-Loading Capacity	Release Kinetics	Injectability	Osteoconductivity	Angiogenic Potential	Immunomodulatory/Senomorphic Capability	References
**Injectable hydrogel**	Natural/synthetic polymers (GelMA, alginate, PEG, collagen)	Weeks to months; enzymatic or hydrolytic	Low stiffness; viscoelastic; not load-bearing	High; encapsulation of small molecules, proteins, EVs, cells	Tunable; burst or sustained release via mesh size or responsive chemistry	Yes	Low unless mineralized	Moderate; supports VEGF delivery and endothelial infiltration	High; supports delivery of senolytics, senomorphics, and immunomodulatory agents	[37,161,162]
**Mineral/ceramic scaffold**	Hydroxyapatite (HA), β-tricalcium phosphate (β-TCP), biphasic HA/TCP	Months to years; dissolution-based	Moderate to high stiffness; brittle; load-bearing in dense forms	Moderate; surface adsorption or pore loading	Sustained but less tunable; dependent on porosity and surface area	No (particulate forms partially)	High; mineral surface supports osteoblast attachment and bone deposition	Low intrinsically; improved with ion doping (Co, Cu, Si)	Low intrinsically; may be functionalized with senolytic-loaded particles or coatings	[[163], [164], [165]]
**Cryogel**	Natural/synthetic polymers (gelatin, HA, MXene composites)	Weeks to months; enzymatic or hydrolytic	Sponge-like; compressible; shape-memory; not load-bearing	High; macroporous architecture supports cell and drug loading	Tunable; responsive to mechanical compression or environmental stimuli	Injectable in compressed form	Moderate; improved with mineral incorporation	Moderate; macroporosity supports vascular infiltration	High; compatible with senolytic, EV, and growth factor co-delivery	[38]
**Composite scaffold**	Combination of polymer + ceramic or polymer + bioactive glass, etc.	Variable; component-dependent	Intermediate; tunable by composition ratio	High; dual-phase loading of hydrophilic and hydrophobic agents	Biphasic or sequential release possible	Dependent on formulation	Moderate to high; mineral phase provides osteoconductive surface	Moderate to high; bioactive glass ions promote angiogenic signaling	Moderate to high; platform flexibility supports multi-agent delivery	[36,166,167]

#### 4.2.1 Scaffold design principles and osteoconductive strategies

Scaffolds serve as engineered 3D constructs that are designed to mimic features of the ECM to support cell attachment, tissue ingrowth, and bone regeneration. Through defined pore size and interconnectivity, scaffolds regulate cell adhesion, migration, and osteogenic differentiation while facilitating vascular infiltration and oxygen diffusion [168]. With scaffolds, characteristics such as surface chemistry and ligand presentation govern adhesion and integrin signaling; stiffness, viscoelasticity, and architecture influence mechano-transduction and lineage commitment; pore interconnectivity and degradation regulate vascular invasion and tissue remodeling; and affinity, mesh size, or responsive chemistries determine therapeutic retention and release [169]. In irradiated bone, these design variables must additionally be judged by whether they preserve cell survival, restore osteogenic-angiogenic coupling, normalize inflammatory and oxidative states, and remain functional within fibrotic, hypoxic, and poorly vascularized tissue. Scaffolds may be fabricated as hydrogels, cryogels, nanofibrous matrices, particulate systems, ceramics, or 3D-printed constructs and may incorporate natural polymers, synthetic polymers, calcium-phosphate bioceramics, or composite combinations [170]. These material classes offer different advantages in cell interaction, mechanics, and degradation for osteogenic regeneration.

These biomaterials typically achieve osteoconduction by providing a mineral-compatible architecture that guides new bone deposition along the scaffold framework, whereas osteoinduction may arise from intrinsic material properties or through incorporation of bioactive signals, such as bone morphogenetic proteins (BMPs), platelet-derived factors, and other osteogenic cues [171]. Osteoconductive scaffolds are most classically represented by calcium-phosphate ceramics. Hydroxyapatite (HA) and β-tricalcium phosphate (β-TCP), alone or combined, provide a mineral surface chemically similar to native bone that supports osteoblast attachment and guided bone deposition. Researchers using biphasic HA/β-TCP blocks implanted in rabbit mandibles promoted new bone formation along the graft surface, directly demonstrating osteoconduction in a craniofacial model [163]. Combining HA and TCP within microsphere- or 3D-printed scaffolds has similarly been shown to support osteoblastic differentiation of marrow stromal cells and new bone formation across multiple animal models without inflammatory reaction, reinforcing HA/TCP composites as a baseline osteoconductive platform on which more active functions are later layered [172].

Biomaterials can also be engineered to actively promote angiogenesis, the process by which new blood vessels sprout from existing vasculature to supply oxygen, nutrients, and circulating progenitor cells to healing tissue. Through inclusion of elements such as silicon, biomaterial scaffolds have been shown to promote angiogenic signaling in bone regeneration [166,173,174]. Researchers have demonstrated that cobalt- and copper-doped bioactive glasses release ions that stabilize HIF-1α and upregulate VEGF in bone marrow stromal and endothelial cells, enhancing vascular ingrowth without added growth factors or cells [164]. Osteoconductive ceramics, bioactive polymers, mineral components, and ion-modified materials illustrate the tissue engineering approach that modifies composition, architecture, bioactive modification, cellular infiltration, and physical cues to promote osteoconduction, osteoinduction, osteogenesis, and vascularization (Fig. 4).

**Fig. 4:**
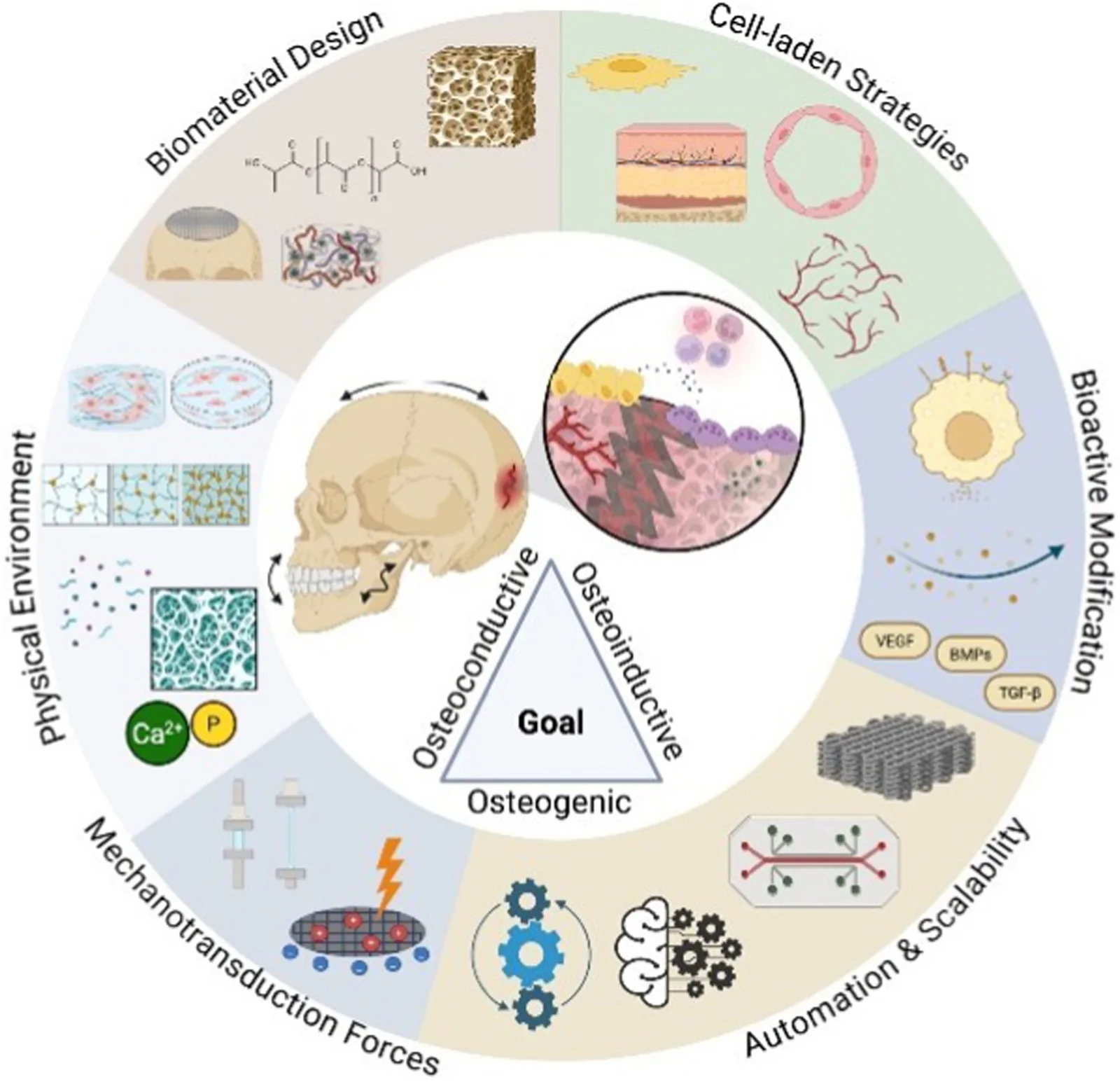
**Regenerative tissue engineering strategies for bone repair.** Overview of regenerative bone tissue engineering approaches. These strategies promote osteoconductivity, osteoinductivity, osteogenicity, and vascularization. Adapted from Ref. [175] under CC BY 4.0.

For ORN, the demands of the implanted material change fundamentally. Materials can no longer act as passive implants; they must modulate the irradiated niche, support repair despite vascular and cellular compromise, and deliver reparative therapeutics. Radiation-specific biomaterials increasingly exemplify this transition from passive osteoconduction to active microenvironmental regulation. Chen et al. developed taurine-loaded covalent organic frameworks (Tau-COFs) for post-radiation jaw regeneration [176]. The platform improved osteogenic outcomes, reduced TRAP-associated osteoclast activity, increased M2-associated macrophage responses, and demonstrated antibacterial activity against *Staphylococcus aureus* and *Prevotella intermedia*. These combined effects position Tau-COF as a multifunctional platform that simultaneously addresses osteogenesis, osteoimmunity, remodeling, and microbial burden within the irradiated niche.

Additional biomaterial modifications allow for radiation-specific regeneration targeting. Wang et al. developed ceria-doped silicate nanozymes that combined SOD/CAT-like ROS elimination with mitophagy restoration; encapsulated DL-3-n-butylphthalide provided pH-responsive proangiogenic signaling, whereas silicate-derived ions supported osteogenesis [177]. Wu et al. functionalized HA particles by lyophilizing lipofectamine-agomiR-34a complexes onto the mineral surface, creating an osteoconductive carrier capable of locally delivering miR-34a to radiation-impaired BMSCs [178]. *In vitro*, the construct increased osteogenic differentiation and expression of RUNX2, BMP2, ALP, and osteocalcin after irradiation. *In vivo*, implantation into 3 mm defects in rat tibiae previously exposed to 15-Gy increased new-bone formation, bone-volume fraction, and cortical continuity relative to control constructs; local miR-34a activity persisted for several weeks. The study illustrates how a familiar mineral scaffold can become a localized nucleic-acid delivery platform, but it represents an irradiated long-bone study rather than an ORN model, and vascular, inflammatory, osteoclastic, and biomechanical outcomes were not established.

The importance of developing scaffolds specifically for irradiated tissues was further demonstrated by Hussein et al. [179]. In this study, critical-sized, full-thickness mandibular defects were created in irradiated and nonirradiated rabbits (Fig. 5Ci-ii) and filled with Bio-Oss, a particulate bovine-derived bone graft. Bio-Oss served as a passive osteoconductive xenograft that provided mineral surfaces for host bone deposition but did not contain therapeutic cells or factors designed to reverse radiation injury. After eight weeks, irradiated defects contained significantly less newly formed bone, more soft tissue, and less defect bridging than identically grafted nonirradiated defects. Thus, although the graft supported bone formation in a healthy environment, it did not overcome the impaired cellularity, vascularity, and regenerative capacity of irradiated bone.

**Fig. 5:**
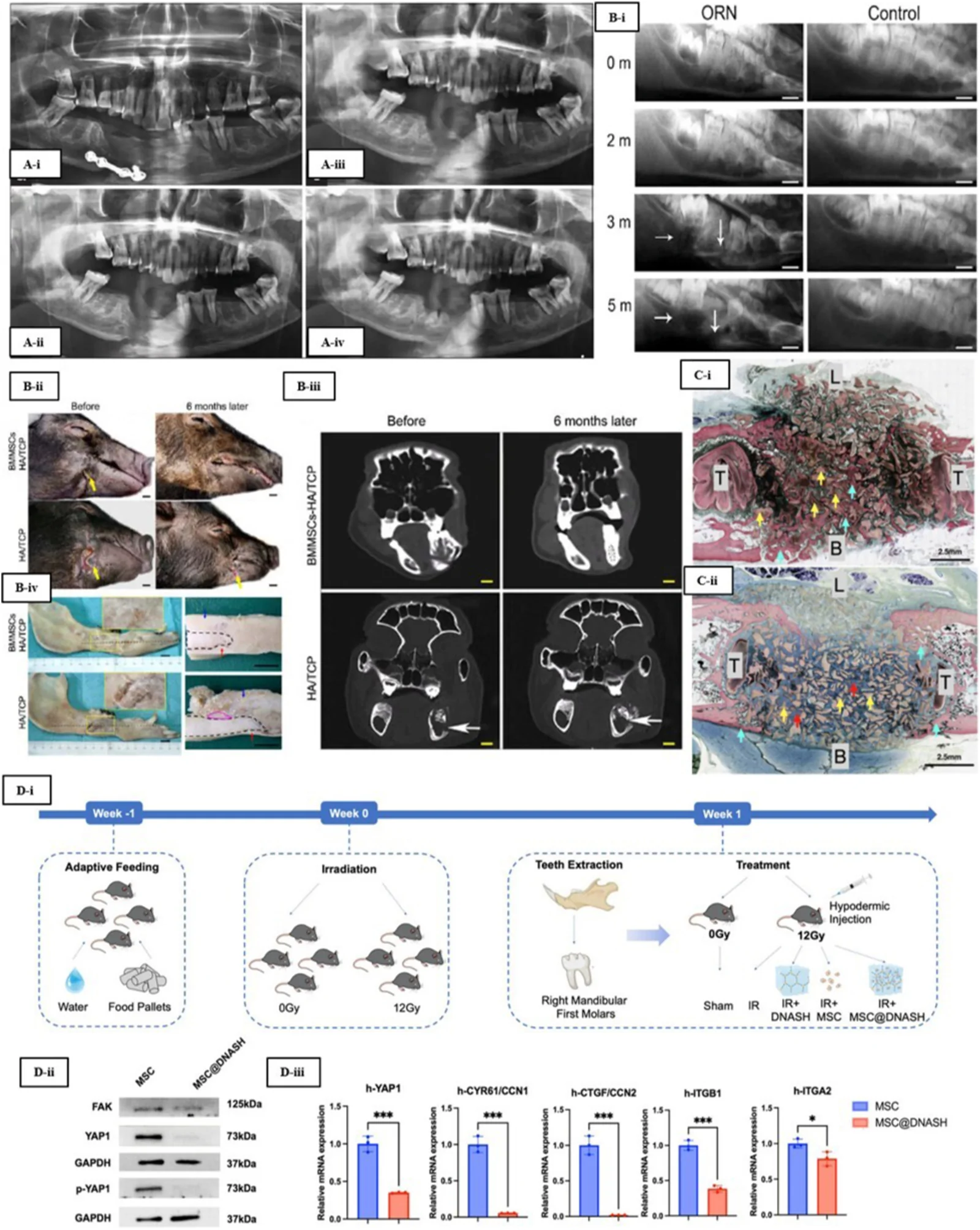
**Clinical and pre-clinical tissue engineering approaches for mandibular ORN.** (A) Serial postoperative radiographs showing progressive bone formation, union, and remodeling at (i) three months, (ii) six months, (iii) twelve months and (iv) two years after osseous tissue engineering treatment. (B) Swine model of ORN following irradiation and tooth extraction: (i) progressive mandibular destruction; (ii-iv) improved soft-tissue healing and bone reconstruction after transplantation of BMSC-HA/TCP compared with HA/TCP alone. Scale bars: 1 cm. (C) Representative histomicrographs of (i) nonirradiated and (ii) irradiated rabbit mandibular defects treated with Bio-Oss, showing newly formed bone and soft-tissue infiltration. T = tooth; B = buccal; L = lingual [179]. (D) DNA supramolecular hydrogel treatment: (i) experimental model of irradiation, molar extraction, and treatment; (ii-iii) effects of 3D hydrogel culture on FAK/YAP1 signaling and mechanosensing-related gene expression in MSCs via Western blot (ii) and RT-PCR analysis (iii). Data are mean ± SD (n = 3); *p < 0.05, **p < 0.01, ***p < 0.001. (A) Reproduced from Ref. [180] under CC BY-NC-SA 4.0. (B) Reproduced with permission from Refs. [181,182] under the STM Permissions Guidelines. (C) Reproduced with permission from Ref. [179] Wiley & Sons. (D) Reproduced with permission from Ref. [37], John Wiley & Sons.

Biomaterials can be used as a localized delivery system. Zheng et al. used GelMA to locally deliver vitamin-D3-induced tolerogenic dendritic cells, improving regeneration of irradiated mandibular defects while directly testing osteoimmune reprogramming [64]. Cheng et al. engineered Fe-doped VOx nanozymes with catalase-, superoxide-dismutase-, and glutathione-peroxidase-like activities to scavenge excess reactive oxygen and nitrogen species, restore mitochondrial function and mitophagy, and provide photothermal antibacterial activity in an ORN of the jaw (ORNJ) model [183]. Chen et al. subsequently developed Mn/Ca diatomic-site nanozymes coated with hybrid membranes derived from M2 macrophages and osteogenically induced human-BMSCs to mimic elements of the jaw vascular unit [184]. The resulting platform combined triple-enzyme redox activity with preferential cellular interaction, M2-associated immune reprogramming, osteogenic support, and improved *in vivo* bone and mucosal repair.

More advanced scaffold systems are designed to respond dynamically to their local environment or to external stimuli. Stimuli-responsive biomaterials can undergo changes in swelling, structure, degradation, or therapeutic release in response to factors such as pH, temperature, enzymatic activity, light, or mechanical loading. Lei et al., for example, developed a thermoresponsive PEG-PLGA-PNIPAM hydrogel that gelled at physiological temperature and incorporated aspirin together with mesoporous silica nanoparticles carrying microRNA-222 for localized delivery [161]. Local sustained co-delivery in a rat mandibular defect enhanced bone regeneration and neural differentiation. Resina et al. similarly engineered an electrical-chemical responsive hydrogel that releases chloramphenicol (CAM) in response to electrical stimulation and pH within the body [162]. These systems provide greater spatial and temporal control over regenerative signaling and can provide benefits in complex craniofacial defects, where inflammatory, vascular, and osteogenic requirements change throughout the healing process.

Scaffolds function not only as passive structural supports but also as active regulators of regeneration by providing spatial and temporal control over mechanical and biochemical cues [168]. They provide appropriate mechanical and architectural support, promote vascular and cellular infiltration, guide osteogenic tissue formation, and serve as a reservoir for regenerative cells or signaling molecules. In the context of ORN, the osteonecrotic environment becomes an essential consideration. Regeneration must occur within a hypovascular, hypocellular, and chronically damaged tissue environment; therefore, scaffold-based strategies for ORN must focus not only on reconstructing lost bone, but also treating the surrounding environment.

#### 4.2.2 Scaffolds as delivery models

Scaffolds can be utilized as a carrier of therapeutics as well as intact regenerative cells. As previously mentioned, MSC therapies are often limited by poor retention, variable survival, and unpredictable biodistribution, particularly within irradiated tissue [185]. Loading cells within a biomaterial matrix may address these limitations by retaining cells at the treatment site while providing adhesive, mechanical, osteoconductive, and biochemical cues that support their survival and regenerative activity. Accordingly, several preclinical and early clinical ORN studies have combined MSCs or other progenitor populations with calcium-phosphate ceramics, hydrogels, platelet-derived matrices, and other scaffold systems.

Mendonça and Juiz-Lopez reported the treatment of three patients with severe craniofacial defects, including one patient with stage IIIR maxillary and mandibular ORN and a pathological mandibular fracture [186]. They started by obtaining 100 mL of heparinized bone marrow aspirate from the posterior ilium, which produced a culture-expanded mixture of early mesenchymal, hematopoietic, and endothelial stem and progenitor cells. The cells were then added into an autologous fibrin- and platelet-rich clot and mineral base of β-TCP and HA. In the patient with ORN, the necrotic tissue was surgically excised, and the mandibular fracture was stabilized with a reconstruction plate. The cellular preparation was placed within the reconstructed region and inoculated into the surrounding skin and muscle, tissue adjacent to the facial nerve, and the remaining necrotic vascular stumps before wound closure. During follow-up, early bone formation, recovery from alveolar nerve anesthesia, facial nerve reinnervation, skin regeneration, and subsequent oral rehabilitation with dental implants and a fixed prosthesis was reported. However, because the cells were administered together with extensive surgical debridement, fracture stabilization, autologous platelet- and fibrin-rich preparations, and calcium phosphate biomaterials, the specific contribution of the transplanted cells could not be determined [186].

Within the ORN environment, early clinical reports remain limited and heterogeneous. Manimaran et al. described the treatment of two patients with mandibular ORN that had not responded to conventional treatment [187]. In the first patient, autologous bone marrow aspirate concentrate was injected around a persistent nonhealing extraction socket and into the adjacent periosteal tissues, using a similar approach to that described by Mendonça and Juiz-Lopez. At the same time, because bone marrow aspirate concentrate contains a heterogeneous mixture of cells and soluble factors, its effects cannot be attributed to stem cells alone. The second patient received culture-expanded allogeneic dental pulp stem cells combined with β-TCP and platelet-rich plasma (PRP). Dental pulp stem cells are neural crest-associated mesenchymal progenitors with osteogenic and angiogenic properties that make them relevant to craniofacial tissue repair. In both patients, progressive soft-tissue closure and radiographic evidence of bone formation beginning approximately two months after treatment was reported. Importantly, healing was evaluated primarily through clinical examination and panoramic radiography, which cannot provide the same quantitative assessment of bone volume, mineral density, or microarchitecture as CT or histological analysis.

Controlled animal studies have delivered more insight into the conditions under which transplanted MSCs may support ORN regeneration. Jin et al. investigated BMSCs in a rat mandibular ORN model done using 30-Gy irradiation followed by molar extraction [188]. MSCs, BMP-2, or their combination were delivered via a hydrogel either immediately after extraction or four weeks later, following the development of ORN. The hydrogel acted as a local carrier for the cells and growth factor, while BMP-2 supplied an osteoinductive signal capable of promoting osteogenic differentiation. Immediate treatment with MSCs alone did not significantly improve bone volume or mineral density, which indicated that the presence of transplanted cells was not sufficient to overcome the acute radiation-damaged environment. In contrast, after ORN had become established, the combined delivery of MSCs and BMP-2 significantly increased both bone volume and mineral density. The superior response to the combination treatment suggests that transplanted MSCs may require strong osteoinductive stimulation to maintain or recover their bone-forming activity within irradiated tissue. It also indicates that the principal benefit of cell therapy may arise from cooperation among the cells, delivery matrix, and locally presented regenerative signals rather than from cellular transplantation alone.

The influence of treatment timing was further evidenced by Park et al. using tonsil-derived MSCs in a rat model of mandibular ORN [140]. Rats received 20-Gy mandibular irradiation followed by tooth extraction, and the MSCs were delivered in Matrigel either immediately after extraction or four weeks later. Immediate MSC administration resulted in greater bone regeneration and higher bone mineral density (Fig. 3A) than delayed administration. This suggests that MSCs may be most effective when delivered during the early healing period, before extensive tissue necrosis, fibrosis, chronic inflammation, and vascular loss become established. At this stage, transplanted cells may preserve the viability of the extraction socket, support residual host progenitor cells, and modulate the initial inflammatory response. Once mature ORN has developed, however, the severely compromised tissue environment may reduce MSC survival and limit their access to oxygen, nutrients, and instructive signals.

In a recent study, Kanakaraj et al. treated six patients with mandibular ORN using a composite osseous tissue engineering strategy following sequestrectomy, alveolectomy, and curettage of the necrotic bone [180]. The regenerative construct contained culture-expanded autologous dental pulp stem cells, bone marrow aspirate concentrate, and PRP, which were loaded into either a β-TCP or HA sponge scaffold and implanted within the mandibular defect. In this construct, the ceramic sponge served as the principal scaffold, while PRP supplied a fibrin-rich phase and concentrated growth factors that helped retain and biologically support the implanted cells. When the debrided mandible remained mechanically unstable, a reconstruction plate was used for fixation, which showed that the ceramic sponge was not intended to provide load-bearing stabilization. Patients reported reduced pain and improved jaw mobility, oral function, and occlusion, with radiographic evidence of mandibular regeneration (Fig. 5A) and no major complications during a mean follow-up of 28 months. Similarly, Chen and Chang placed platelet-rich fibrin (PRF) within surgically debrided maxillary and mandibular ORN sites [189]. PRF is a second-generation platelet concentrate in which platelets, leukocytes, cytokines, and growth factors are entrapped within a dense autologous fibrin network. This network acts as a resorbable provisional ECM that supports clot stability, cell migration, soft-tissue closure, and sustained local growth-factor release, but it is not a rigid, defect-spanning bone scaffold. In the mandibular lesion, the mucosa healed within three weeks, and radiographic bone healing was observed after ten months.

More substantial mechanistic support for scaffold-assisted ORN regeneration has been obtained in animal models. Wang et al. developed advanced mandibular ORN in miniature pigs using a single 25-Gy radiation dose followed by tooth extraction (Fig. 5Bi) [181]. Following removal of necrotic bone and soft tissue, autologous BMSCs were implanted using HA/β-TCP particles as the carrier. While the ceramic material provided an osteoconductive mineral, its particulate structure allowed it to conform to the irregular debrided defect and retain the transplanted cells locally. Treatment with the cell-seeded HA/β-TCP carrier resulted in the regeneration of bone, microvessels, and surrounding soft tissue, together with resolution of the ORN-associated fistula (Fig. 5Bii-Biv). In contrast, the ceramic carrier on its own did not produce comparable regeneration without the cellular component.

In another animal model, Janus et al. examined a softer, injectable scaffold system in an athymic rat model of mandibular ORN [190]. Following 20-Gy irradiation and molar extraction, the animals received saline, human adipose-derived MSCs, a PRP-collagen formulation, or adipose-derived stem cells combined with PRP and collagen. In this system, collagen provided a natural ECM-like carrier, while PRP supplied a fibrin-forming component and soluble regenerative factors. Unlike the rigid calcium phosphate scaffolds used in other studies, the PRP-collagen formulation was an injectable biologic matrix intended primarily to retain cells and signaling molecules at the extraction site rather than maintain mandibular geometry or withstand mechanical loading. Adipose-derived stem cell administration increased mandibular bone volume by as much as 36%, while PRP-collagen, with or without stem cells, increased osteoblast and reduced osteoclast numbers, respectively. This demonstrates that even a temporary collagen-based matrix may support cellular and paracrine therapies in ORN, although increased fibrosis following stem-cell administration and the relatively short follow-up period demonstrates the need for further optimization.

More recently, Li et al. developed an injectable, shear-thinning DNA supramolecular hydrogel as a 3D scaffold for MSCs in a murine jaw model of ORN (Fig. 5Di) [37]. In contrast to passive ceramic carriers, this hydrogel was designed to not only retain cells but also to regulate their behavior. The DNA-based network prolonged the local retention of transplanted MSCs and their exosomes for more than three weeks, which addressed the rapid cellular dispersion and clearance that commonly limit cell therapy, as well as time-dependent release. The mechanical and biochemical environment created by the hydrogel altered FAK-integrin signaling, which links cell adhesion to intracellular mechanotransduction, and Hippo-YAP signaling, a mechanosensitive pathway regulating cell proliferation, survival, and differentiation. Together, these signaling shifts drove MSC exosomal microRNA and paracrine profiles toward a more regenerative phenotype (Fig. 5Dii-iii). Treatment with the MSC-loaded hydrogel caused removal and remodeling of necrotic bone, stimulated osteogenesis, reduced inflammation, and restored mandibular architecture. These results demonstrate that scaffolds can function as regulators of mechanotransduction and cell secretion.

#### 4.2.3 Pro-angiogenic biomaterial strategies for vascularized bone regeneration

Restoration of vascular supply is a central requirement for bone regeneration in ORN, where radiation-induced endothelial injury produces a persistently hypovascular, hypoxic microenvironment that limits progenitor cell survival, osteogenic-angiogenic coupling, and therapeutic engraftment. Biomaterial strategies targeting angiogenesis therefore represent a critical complement to osteogenic and senotherapeutic approaches.

Growth factor delivery is the most extensively explored pro-angiogenic strategy in bone tissue engineering [145]. VEGF remains the primary target, with scaffolds designed to provide sustained local release to promote endothelial sprouting and neovascularization. Sequential or dual delivery of VEGF alongside angiopoietins or bone morphogenetic proteins has been shown to improve both vascular maturation and osteogenic outcomes in preclinical bone defect models, reflecting the coordinated nature of angiogenic-osteogenic coupling during repair [166]. In irradiated tissue, where endogenous VEGF signaling is suppressed and vascular density is reduced, localized growth factor delivery may help restore the angiogenic threshold required to sustain regeneration [71,72].

Co-delivery or co-culture of endothelial progenitor cells with MSCs represents a complementary cellular strategy [70]. Endothelial progenitor cells contribute directly to neovascularization while MSC-derived paracrine signals support endothelial survival and tube formation. Biomaterial carriers that support the co-culture of these populations, maintaining cell viability, spatial organization, and paracrine crosstalk within the irradiated niche, are therefore of particular interest for ORN applications. Pre-vascularized scaffold designs, in which endothelial networks are established within the construct prior to implantation, offer a further strategy to accelerate tissue integration and blood vessel ingrowth within the poorly perfused irradiated niche [191].

At the microenvironmental level, endothelial senescence represents an underappreciated contributor to vascular failure in ORN. Irradiation induces senescence in endothelial cells, impairing their angiogenic capacity and driving SASP-mediated paracrine dysfunction that propagates vascular injury to neighboring cells [69,70]. Senomorphic strategies targeting NF-κB and JAK/STAT signaling within senescent endothelial populations may therefore partially restore angiogenic competence, and biomaterial platforms capable of delivering senomorphics alongside pro-angiogenic signals represent a logical next step for the field [192,193]. Connecting these vascular strategies back to the radiation-induced endothelial injury described in Section 2.1.6, it is clear that durable ORN regeneration will require not only structural bone repair but concurrent restoration of the vascular niche.

## 5 Cellular senescence-targeted therapeutic strategies in ORN

Accumulating evidence implicates cellular senescence as one of the central mechanisms underlying the chronicity and treatment resistance of ORN. Cellular senescence is defined by irreversible cell-cycle arrest accompanied by sustained metabolic activity and SASP secretion (Fig. 6A) [31,194]. Upstream DNA damage and oncogenic stress converge on p53/p21 and p16/pRB checkpoint pathways, with downstream NF-κB and mTOR activation driving the broad SASP transcriptional program responsible for paracrine tissue dysfunction. In the context of RT, IR generates excessive oxidative stress and extensive genomic injury, activating DNA damage response pathways that drive SIPS across multiple bone-resident cell populations [[26], [27], [28],^34^].

**Fig. 6:**
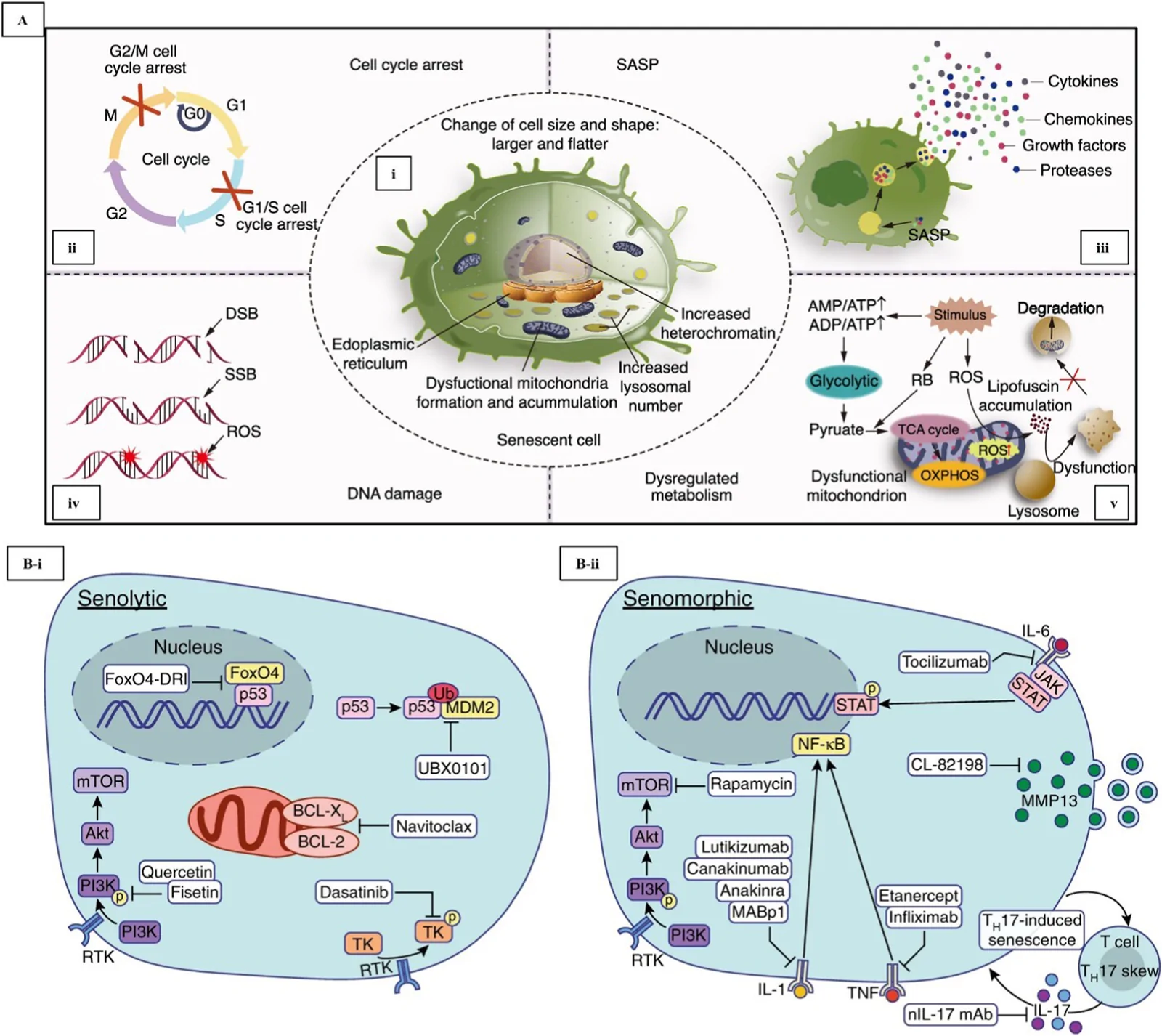
**Hallmarks and therapeutic targeting of cellular senescence.** (A) Major features and causes of cellular senescence: (i) enlarged, flattened morphology with nuclear and organelle dysfunction; (ii) stable cell-cycle arrest, mostly in the G1 phase; (iii) secretion of cytokines, chemokines, growth factors, and proteases, which comprise the SASP; (iv) persistent DNA damage; and (v) metabolic dysregulation, mitochondrial dysfunction, ROS production, and lipofuscin accumulation [195]. (B) Senescence-targeting therapies: (i) senolytic strategies selectively induce apoptosis in senescent cells by targeting prosurvival pathways and receptor tyrosine kinase signaling; and (ii) senomorphic strategies suppress SASP production or signaling using agents such as MMP13 and mTOR inhibitors or antibodies targeting IL-1, IL-6, TNF, and IL-17A/F. Ub = ubiquitin [196]. (A) Reproduced from Ref. [195] under CC BY 4.0. (B) Reproduced from Ref. [196] under CC BY 4.0.

Senescent cells persist in a hyporeplicative yet metabolically active state and are characterized in part by the expression of the cell-cycle inhibitor p16^INK4a^, which enforces cell-cycle exit and prevents uncontrolled proliferation [30,31]. While this response initially serves as a tumor-suppressive mechanism, pathological accumulation of senescent cells drives sustained inflammation through SASP factor release, induces bystander senescence in neighboring cells, and undermines tissue homeostasis [32,33]. SASP signaling is reinforced through autocrine and paracrine feedback loops, leading to self-propagating amplification of senescence-associated dysfunction within irradiated tissues [34].

Bone tissue is particularly vulnerable to senescence-driven degeneration and decline [197]. Radiation-induced senescence in bone is regulated primarily through p53/p21 and p16/pRB signaling pathways [83,198,199]. Importantly, p21-positive senescent cells predominate during the acute phase following irradiation and contribute to early bone loss, whereas p16-positive senescent cells accumulate more slowly and drive long-term skeletal degeneration [51,56,200,201]. This temporal distinction provides a mechanistic framework for understanding the delayed onset and progressive nature of ORN following RT.

In attempts to target and treat cellular senescence, researchers have shown that IR induces a persistent senescent phenotype characterized by elevated SA-β-galactosidase (SA-β-gal) activity, sustained DNA damage signaling, and dysregulation of senescence-associated gene networks, effects that are challenging to reverse using conventional metabolic or growth factor-based interventions [202]. However, experimental clearance or suppression of senescent cells has demonstrated therapeutic benefit. In aged (20- to 22-month-old) mouse models with established bone loss, activation of the INK-ATTAC caspase-8 (senescent pathway) or treatment with senolytic agents/JAK inhibitors resulted in increased bone mass, improved microarchitecture, and enhanced mechanical strength [203].

Cellular senescence can arise through replicative exhaustion or stress-induced mechanisms. The Hayflick limit describes the finite replicative capacity of human somatic cells, which undergo approximately 50 population doublings before entering replicative senescence due to telomere attrition [204]. Telomeres, guanine-rich repetitive sequences maintained by the shelterin complex, shorten with each cell division in the absence of telomerase activity, ultimately triggering DNA damage responses mediated by ATM/ATR signaling and downstream activation of p53, p21CIP1, and p16^INK4a^ [[205], [206], [207]].

Importantly, radiation accelerates senescence through telomere-independent mechanisms. Telomeric regions are particularly susceptible to oxidative damage, and IR induces telomere-associated DNA damage foci even in cells with adequate telomere length [208,209]. This form of radiation-induced cellular senescence (RICS) converges on the same checkpoint pathways as replicative senescence but occurs independently of cumulative cell divisions [210]. Clinically, RICS is a double-edged sword: while limiting proliferation of damaged cells may suppress tumorigenesis, persistence of senescent cells impairs regeneration and accelerates tissue degeneration in irradiated bone [195].

### 5.1 Senotherapeutic strategies

It is important to note that direct evidence for senotherapeutic intervention in mandibular ORN remains absent. The strategies discussed below derive from *in vitro* studies of irradiated bone-resident cells, small-animal models of focal limb or femoral irradiation, and aging-related bone loss models—none of which fully recapitulate the complex microenvironment of fractionated radiation-induced mandibular ORN. Where evidence is extrapolated from non-ORN systems, this is noted explicitly. These findings nonetheless provide a mechanistic rationale for translating senotherapeutic strategies to ORN, and represent a compelling basis for future investigation in clinically relevant mandibular models. Senotherapy encompasses a class of therapeutic approaches designed to mitigate the pathological consequences of senopathies (diseases associated with cellular senescence) [[211], [212], [213]]. These strategies are broadly divided into senolytics, which selectively induce apoptosis in senescent cells (Fig. 6Bi), and senomorphics, which attenuate SASP-mediated inflammation and fibrosis without eliminating the senescent population (Fig. 6Bii) [194].

Currently, clinical experience with senotherapy in humans remains limited, and most available evidence derives from *in vitro* and *in vivo* studies. In these preclinical settings, senotherapeutic interventions have been shown to reduce chronic inflammation, improve tissue repair, and attenuate functional decline across multiple age- and injury-related pathologies [203,214,215]. Table 4 outlines senotherapeutic approaches for RICS that may be translatable to ORN pathophysiology.

**Table 4:** Senescence-targeting therapeutic strategies relevant to radiation-induced tissue injury and ORN.

Senotherapeutic Strategy	Core Molecular Targets	Representative Example(s)	Expected Therapeutic Benefit in ORN	Major Risks and Constraints	References
Senolytics	Anti-apoptotic survival pathways enriched in senescent cells (e.g., BCL-2 family, PI3K/AKT, FOXO)	Dasatinib + quercetin (D + Q); navitoclax; fisetin	Selective removal of pathological senescent cells to restore bone turnover, angiogenic capacity, and tissue remodeling	Off-target toxicity; thrombocytopenia and hematologic effects; potential impact on non-senescent tissue	[194,212,[216], [217], [218], [219]]
Senomorphics	NF-κB, JAK/STAT, mTOR signaling pathways regulating inflammatory and profibrotic SASP components	Ruxolitinib (JAK inhibitor); rapamycin	Attenuation of chronic inflammatory milieu and fibrotic signaling while preserving growth arrest; reduction of bystander senescence	Requires continuous or long-term administration; incomplete reversal of senescent burden	[192,193,212,220,221]
Mitochondrial and redox reprogramming	ROS scavenging; mitochondrial quality control and metabolic reprogramming	Chondroitin sulfate methacryloyl (ChSMA) composite hydrogel scaffold incorporating tea polyphenol-reduced graphene	Reduction of radiation-induced oxidative stress and senescence triggers; partial restoration of cellular function	Limited specificity for senescent cells; primarily senomorphic rather than senolytic	[[222], [223], [224], [225]]
Immune-mediated senescent cell clearance	Activation of endogenous immune surveillance pathways, including natural killer (NK) cells and macrophage-mediated phagocytosis	NK-cell activation; macrophage-mediated phagocytosis	Restoration of endogenous clearance of senescent cells; potential normalization of inflammatory milieu	Immune dysfunction in irradiated tissues; impaired immune competence in cancer patients	[[226], [227], [228], [229], [230]]
Epigenetic regulation	Chromatin remodeling and senescence-associated transcriptional networks	Histone deacetylase (HDAC) inhibitors; chromatin modifiers	Destabilization or partial reprogramming of the senescent state; modulation of SASP expression	Broad epigenetic effects; limited cell-type specificity; oncogenic risk	[[231], [232], [233], [234]]
Regenerative combination approaches	Integrated senescence targeting with osteogenic and angiogenic support	Injectable bone-regenerative scaffold (GelMA hydrogel + mesoporous bioglass) delivering D + Q followed by BMP-2	Synergistic restoration of skeletal structure and function by coupling senescent cell clearance with regenerative cues	Complex delivery strategies; regulatory and clinical trial design challenges	[235]

In a recent study, Chandra et al. provided direct evidence that senolytic therapy can mitigate radiation-induced damage in bone [51]. Four-month-old male C57BL/6 mice received a single 24-Gy focal dose to a 5 mm region of the distal femoral metaphysis, which increased expression of p16^Ink4a^, p21, and SASP genes, and reduced osteoblast and osteocyte numbers. Then, dasatinib (5 mg/kg), quercetin (50 mg/kg), fisetin (50 mg/kg), or D + Q was administered orally on the day of irradiation and again 14 days later. Dasatinib or quercetin alone provided partial protection, whereas D + Q produced the most consistent effects, reducing senescence markers and SASP-factor expression while increasing trabecular bone-volume fraction, connectivity density, osteoblast numbers, mineralization, and bone-formation rate while decreasing marrow adiposity. Fisetin alone did not significantly protect irradiated bone under the dose and schedule tested. However, this does not mean that fisetin lacks activity against irradiated bone cells, as the amount reaching irradiated bone was not quantified. Low bioavailability and rapid systemic clearance represent major pharmacological hurdles limiting the clinical utility of first-generation senolytics [236].

The incorporation of senotherapeutics into bone tissue engineering strategies, in which biomaterials serve not only as structural or regenerative platforms but also as targeted delivery vehicles, holds great promise for the treatment of bone senopathies. For example, Li et al. addressed the pharmacological hurdles of free senolytics by developing alendronate-functionalized liposomes containing D + Q, termed Aln-Lipo-DQ, which had affinity for HA and preferential accumulation in the femur and tibia [237]. In BMSCs that became senescent by doxorubicin or 10-Gy irradiation, liposomal D + Q formulations reduced the proportion of p16-positive (Fig. 7A) and γH2AX-positive (Fig. 7B) cells, and Aln-Lipo-DQ reduced SA-β-gal activity while restoring alkaline phosphatase activity, mineralization, and osteogenic differentiation. In a doxorubicin-induced model of skeletal senescence (Fig. 7C), Aln-Lipo-DQ reduced p16- and p21-positive bone cells and osteoclast abundance while increasing osteocalcin-positive osteoblasts (Fig. 7D). In eight-week-old male C57BL/6 mice exposed to 15-Gy irradiation of the lower limbs, the targeted formulation showed greater improvements in trabecular microarchitecture than orally administered or untargeted liposomal D + Q, increasing bone-volume fraction approximately 2.91-fold relative to untreated irradiated controls, and improved the osteogenic capacity of bone marrow stromal cells isolated from treated animals.

**Fig. 7:**
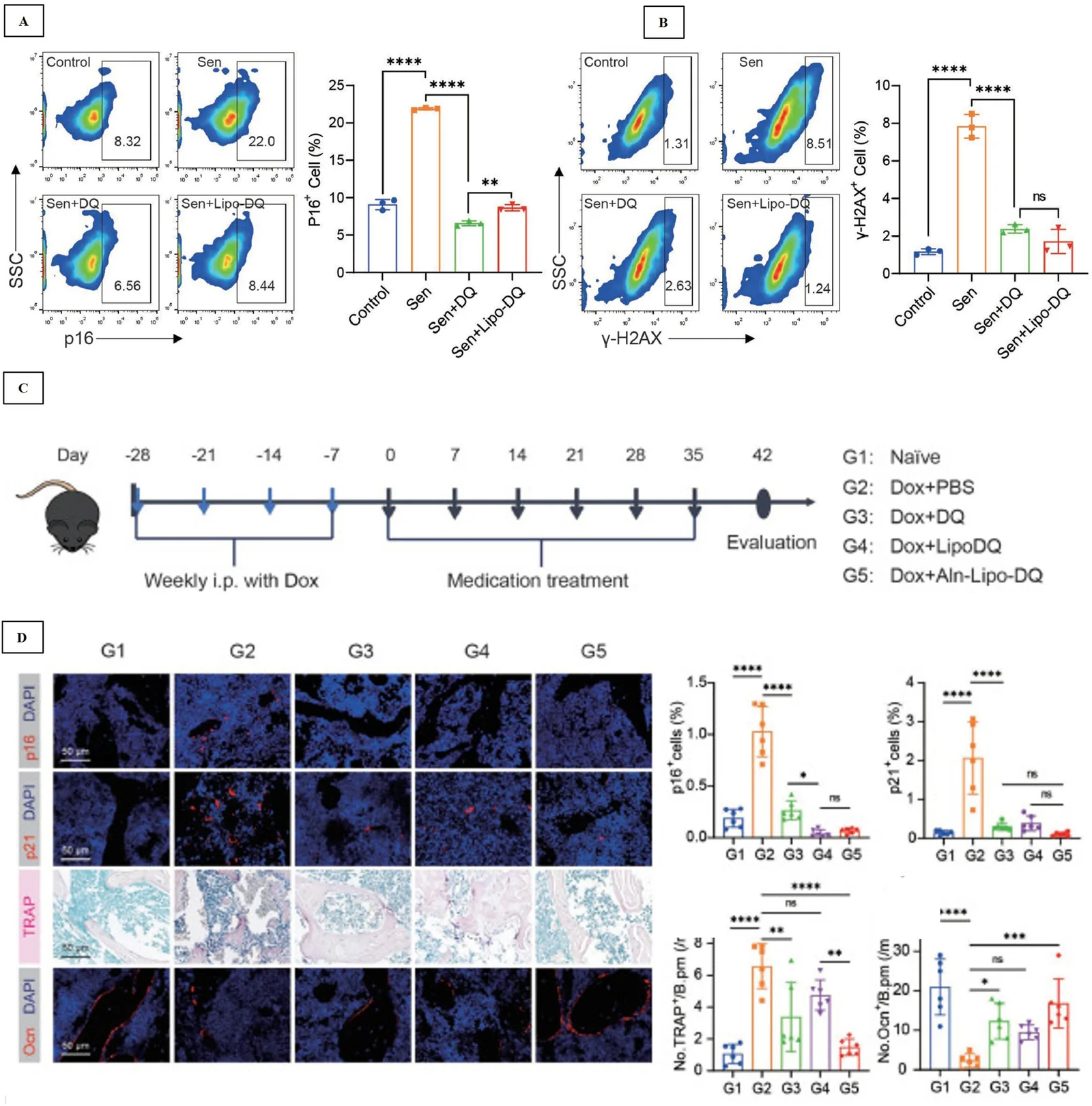
**Bone-targeted senolytic delivery reduces skeletal senescence.** Effects of free D + Q, liposomal D + Q (Lipo-DQ), and alendronate-functionalized liposomal D + Q (Aln-Lipo-DQ): (A, B) flow cytometry quantification of p16-positive and γH2AX-positive senescent BMSCs, respectively (n = 3); (C) timeline of the doxorubicin-induced in vivo model; and (D) immunofluorescence for p16, p21, and osteocalcin (Ocn) and TRAP staining in femurs, with quantification of positive cells per bone perimeter (B.pm) (n = 6). (B) Reproduced with permission from Ref. [238]. (A-B) Adapted from Ref. [165] under CC BY 4.0.

Xing et al. further demonstrated the value of combining senolytics with bone-targeted biomaterials by developing quercetin-loaded liposomes functionalized with the HA-binding peptide (DSS)_6_ [165]. Within senescent bone marrow cells (BMCs), quercetin treatment decreased SA-β-gal activity and the expression of senescence-associated markers while partially restoring cellular proliferation, mineral deposition, and osteogenic differentiation, suggesting that depletion or suppression of the senescent population could recover the function of the remaining osteoprogenitor pool. Because the low bioavailability and broad tissue distribution of systemically administered quercetin could restrict its efficacy and produce off-target effects, the authors encapsulated it within liposomes functionalized with (DSS)_6_ (Fig. 8A–G). This peptide has affinity for low-crystallinity HA, enabling preferential liposome accumulation at actively remodeling mineralized surfaces where senescent skeletal cells are likely to impair bone formation. Compared with free quercetin and non-targeted liposomal formulations, (DSS)_6_-functionalized quercetin liposomes more effectively reduced the burden of senescent cells within bone, restored the proliferative and osteogenic capacity of BMSCs isolated from treated animals, increased osteoblast-associated activity, and improved bone mass and trabecular microarchitecture. Similarly, Tomé et al. improved senolytic specificity by engineering polymeric nanoparticles loaded with navitoclax, a BCL-2/BCL-xL inhibitor, demonstrating targeted senescent cell clearance without the systemic thrombocytopenia associated with free navitoclax (Fig. 8H) [238]. Together, these studies illustrate a complementary strategy to conventional tissue engineering approaches that provide scaffolds, cells, or osteoinductive signals without directly correcting the dysfunctional host environment.

**Fig. 8:**
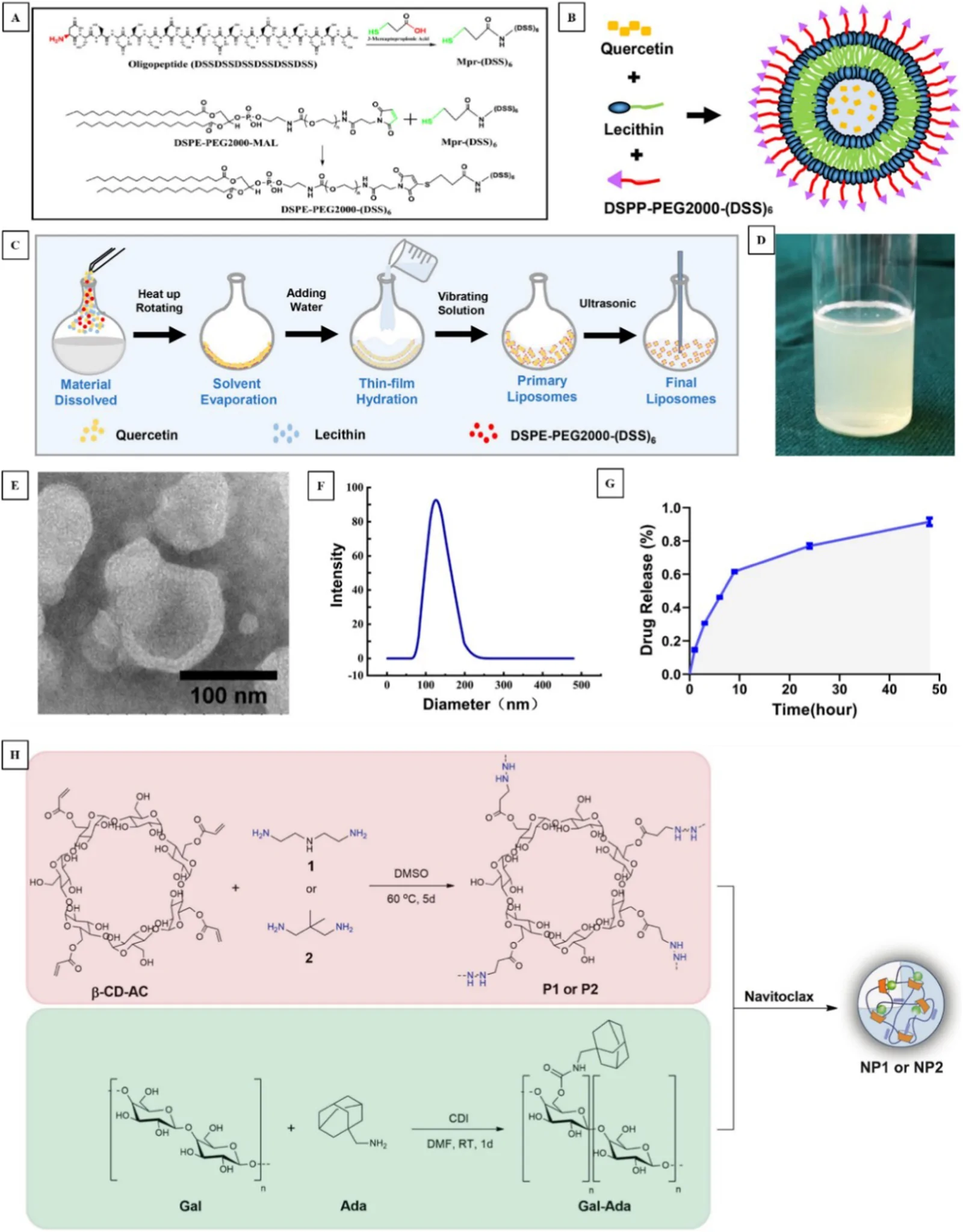
**Bone-targeted and senolytic nanoparticle delivery systems.** (A) Synthetic routes of Mpr-(DSS)_6_ and DSPE-PEG_2000_-(DSS)_6_. (B) Structure of quercetin-loaded bone-targeted liposomes. (C) Preparation of quercetin-loaded bone-targeted liposomes using thin-film ultrasonic dispersion. (D) Uniform suspension of the liposomes in saline. (E) Transmission electron microscopy (TEM) image of the liposomes. Scale bar: 100 nm. (F) Particle size distribution measured by dynamic light scattering. (G) Quercetin release from the liposomes in phosphate-buffered saline (PBS) over 50 h, measured by high-performance liquid chromatography (HPLC). (H) Synthesis of navitoclax-loaded nanoparticles through host-guest interactions between cyclodextrin-based polymers and adamantane-modified galactan. NP1 and NP2 contain different diamine monomers. (A-G) Reprinted from Ref. [165]. Copyright © 2023, with permission from Elsevier. (H) Reproduced from Ref. [239] under CC BY 4.0.

Among the senotherapeutic strategies outlined above, senolytics, particularly D + Q in combination, currently hold the strongest mechanistic rationale for translation to ORN. The convergent evidence linking radiation-induced p21- and p16-positive senescent cell accumulation to progressive bone loss, impaired osteogenesis, and vascular failure directly parallels the pathophysiology targeted by D + Q in preclinical bone models. Senomorphic strategies, particularly JAK/STAT inhibition, represent a complementary rather than competing approach, and may be especially relevant in the ORN context where complete senescent cell clearance may not be achievable in chronically irradiated tissue. Immune-mediated clearance, epigenetic reprogramming, and mitochondrial redox strategies remain earlier in development and require substantially more mechanistic work before their ORN relevance can be assessed. Of all the platforms discussed, biomaterial-assisted local delivery of senolytics (i.e., bone-targeted or injectable scaffold-based systems) represents the most translatable near-term strategy, as it addresses both the pharmacokinetic limitations of systemic senolytic administration and the localized nature of mandibular ORN pathology.

The regenerative strategies discussed in this review are summarized in Table 5, spanning approaches from proof-of-concept *in vitro* irradiated cell models to early human feasibility reports. Across these studies, the therapeutic focus has expanded from replacement of radiation-damaged cells and passive structural support toward restoration of endogenous cellular function and active modification of the irradiated microenvironment.

**Table 5:** Summary of cell-based, biomaterial-assisted, and senotherapeutic regenerative strategies for ORN and radiation-induced bone injury.

Cell Type/Intervention	Model	Carrier	Route	Results	References
Cell Replacement, Paracrine Rescue, and Endogenous-Cell Recovery					
Autologous BMSCs	Wistar rat hindlimb irradiated with 30-Gy; treated 3 months later	___	Labeled BMSCs (2 × 10^6^ cells) were injected in irradiated tibias	∼40% cell retention at 1 week with increased bone blood flow and osteoblastic metabolism; therapeutic effects were transient and diminished by ∼2 months	Phulpin et al. [133]
BMSCs + BMP-2	Rat mandibular ORN; 30-Gy irradiation + molar extraction	Hydrogel	Local delivery immediately after extraction or 4 weeks later	Immediate BMSCs alone did not significantly improve bone volume or mineral density; combined BMSCs + BMP-2 after established ORN significantly increased both	Jin et al. [188]
Tonsil-derived MSCs	Rat mandibular ORN; 20-Gy irradiation + tooth extraction	Matrigel	Local/submucosal delivery immediately after extraction or 4 weeks later	Immediate MSC administration produced greater bone regeneration and higher bone mineral density than delayed administration	Park et al. [140]
Human adipose-derived MSCs	Athymic rat mandibular ORN; 20-Gy + molar extraction	PRP-collagen injectable matrix	Local administration	hAD-MSCs increased mandibular bone volume by up to 36%; PRP-collagen ± cells increased osteoblasts and reduced osteoclasts; increased fibrosis occurred after stem-cell administration	Janus et al. [190]
Human dental pulp stem cell-derived small EVs	Rat ORN of the jaw + irradiated rat bone-marrow cells *in vitro*	Small EVs	Intravenous administration	Reduced senescence-associated gene expression in irradiated cells; increased new bone formation and improved gingival coverage *in vivo*	Ohara et al. [139]
Human amniotic MSC-derived small EVs	Rat jaw ORN + irradiated BMSC and endothelial-cell cultures	Small EVs	Local administration	Improved bone formation and vascular regeneration; restored osteogenic function in irradiated BMSCs and supported endothelial activity; PI3K/AKT signaling implicated	Zhou et al. [141]
BMSC-derived CYR61-containing migrasomes	*in vitro* irradiated BMSCs	BMSC-derived migrasomes carrying CYR61	*In vitro* administration to irradiated BMSCs	Restored migration and osteoblastic differentiation of irradiated BMSCs; increased osteogenic markers and mineralization; integrin αvβ3/ERK signaling implicated	Yan et al. [142]
BMSC-derived exosomes after CeONP preconditioning	Irradiated BMSCs + rat radiation-induced jaw-bone injury	Exosomes isolated from CeONP-preconditioned BMSCs	*In vivo* administration; exact route not specified	Reduced radiation-associated ROS, restored osteogenic differentiation, and improved bone regeneration in irradiated jaw; miR-21-5p/STAT3 implicated	Zhang et al. [146]
CTB-Pro-IGF-1	Rat mandibular ORN; 50-Gy jaw irradiation + secondary trauma	Plant-bioencapsulated CTB-Pro-IGF-1 fusion protein	Oral treatment for 2 weeks	Increased bone volume, mineral density, trabecular thickness and trabecular number; increased viable bone/osteocytes and reduced fibrous healing	Akintoye et al. [148]
Biomaterial-Assisted Regenerative Approaches					
Cell-free xenograft	Critical-sized full-thickness mandibular defects in irradiated vs nonirradiated rabbits	Bio-Oss particulate bovine-derived bone graft	Local implantation	Irradiated defects showed less new bone, more soft tissue, and less defect bridging; passive graft failed to overcome radiation-impaired cellularity and vascularity	Hussein et al. [179]
BMSCs/osteoprogenitor-targeted miR-34a	*In vitro* irradiated BMSCs + rat tibial 3-mm defect after 15-Gy; not ORN	HA particles functionalized with lipofectamine-agomiR-34a	Local implantation into irradiated tibial defect	Increased RUNX2, BMP2, ALP and osteocalcin; increased new bone formation and BV/TV, improved cortical continuity, and sustained local miR-34a availability	Wu et al. [178]
MSCs	Murine ORN of the jaw	Injectable shear-thinning DNA supramolecular hydrogel	Local implantation	Prolonged MSC/exosome retention >3 weeks; altered FAK-integrin and Hippo-YAP signaling and paracrine profiles; promoted necrotic-bone remodeling and osteogenesis, reduced inflammation, and restored mandibular architecture	Li et al. [240]
Autologous BMSCs	Miniature-pig advanced mandibular ORN; 25-Gy + tooth extraction	HA/β-TCP particles	Local implantation after removal of necrotic bone and soft tissue	Regeneration of bone, microvessels, and surrounding soft tissue with resolution of ORN-associated fistula; ceramic carrier alone was inferior	Wang et al. [181]
Tolerogenic dendritic cells in GelMA	Radiation-induced mandibular injury/defect	GelMA	Local cell-delivery matrix	Local tolerogenic-DC delivery improved regeneration while targeting radiation-driven dendritic-cell maturation and BMSC dysfunction	Zheng et al. [64]
Total bone marrow/bone marrow cell extract	Irradiated rat bone defects outside the jaw	60% HA/40% β-TCP macroporous biphasic calcium phosphate	Local scaffold ± total bone marrow; systemic bone-marrow-cell extract also tested	Total bone marrow + scaffold produced greatest local new bone formation; scaffold alone produced little regeneration; systemic extract further improved bone formation	Michel et al. [241]
Microenvironmental Reprogramming					
BMSCs/macrophage and osteoclast modulation	Post-radiation craniofacial bone-regeneration model	Taurine-loaded covalent organic framework (Tau-COF)	Local treatment	Improved osteogenesis, reduced TRAP-associated osteoclast activity, increased M2-associated macrophage responses, antibacterial activity against *S. aureus* and *P. intermedia*, and modulated RANKL/OPG-associated remodeling	Chen et al. [176]
NBP-loaded ceria-doped silicate nanozymes	Preclinical radiation-induced jawbone injury/ORNJ model	Ceria-doped silicate nanozymes encapsulating DL-3-n-butylphthalide (NBP)	*In vivo* administration; exact route not specified in the current manuscript/available abstract	SOD/CAT-like ROS scavenging restored mitophagy; pH-responsive NBP release promoted angiogenesis, while silicate-derived ions supported osteogenesis and jawbone regeneration	Wang et al. [177]
Fe-doped VOx nanozymes (Fe-VOx)	Sprague-Dawley rat ORNJ model; mandibular irradiation followed by molar extraction and *S. aureus* challenge	Fe-doped VOx nanozyme; no separate structural carrier	Local injection (100 μg/mL) for 14 days; ± NIR photothermal treatment	Scavenged RONS through CAT-, SOD-, and GPx-like activity; restored mitochondrial function and mitophagy, improved the immune and regenerative microenvironment, and provided photothermal antibacterial activity	Cheng et al. [183]
JVU-biomimetic Mn/Ca diatomic-site nanozymes (NBP@Mn-CaDSN@HM)	Rat ORNJ model + irradiated-cell mechanistic studies	NBP-loaded Mn/Ca diatomic-site nanozyme coated with hybrid membranes derived from M2 macrophages and osteogenically induced hBMSCs	Targeted *in vivo* administration; exact administration route not specified in the current manuscript	Scavenged RONS, restored mitophagy, suppressed cuproptosis, promoted M2 polarization, osteogenesis, and angiogenesis; improved mucosal healing and increased BV/TV ∼2.3-fold versus irradiated controls	Chen et al. [184]
Rat mandibular BMSCs	*In vitro* irradiated rat mandibular BMSCs		LIPUS	Partially restored cytoskeletal organization, viability, migration, and osteogenic differentiation through RhoA/ROCK modulation	Zhang et al. [149]
D + Q/dasatinib/quercetin/fisetin	4-month-old male C57BL/6 mice; 24-Gy focal distal-femoral irradiation		Oral dosing on day of irradiation and 14 days later	D + Q most consistently reduced senescence/SASP markers, increased BV/TV, connectivity, osteoblasts, mineralization and bone-formation rate, and decreased marrow adiposity	Chandra et al. [51]
Senescent BMSCs; D + Q	*In vitro* BM-MSC senescence + C57BL/6 mice with 15-Gy lower-limb irradiation	Alendronate-functionalized liposomes carrying D + Q (Aln-Lipo-DQ)	Systemic targeted delivery	Reduced SA-β-gal, p16 and γH2AX; restored ALP, mineralization and osteogenesis; increased BV/TV ∼2.91-fold, reduced p16/p21+ cells and osteoclasts, and increased osteocalcin + osteoblasts	Li et al. [237]
Senescent BMCs/BMSCs; quercetin	Senescent BMCs *in vitro* + aged-mouse bone model; not radiation/ORN	Quercetin-loaded liposomes functionalized with HA-binding peptide (DSS)_6_	Targeted systemic delivery	Reduced SA-β-gal and senescence markers; restored proliferation, mineral deposition and osteogenic differentiation; improved bone mass and trabecular microarchitecture	Xing et al. [165]
Human Feasibility					
Mixed stem/progenitor cells (mesenchymal, hematopoietic, endothelial)	Human severe craniofacial defects; one patient with stage IIIR maxillary/mandibular ORN and pathologic fracture	Autologous fibrin- and platelet-rich clot with β-TCP + HA mineral base	Local placement into reconstructed region and surrounding tissues after debridement/fixation	Early bone formation, nerve recovery, skin regeneration, and later implant-supported oral rehabilitation	Mendonça & Juiz-Lopez [186]
Autologous bone marrow aspirate concentrate (BMAC)	Human mandibular ORN refractory to conventional treatment (patient 1 of 2)	3 mL BMCs with β-tricalcium phosphate/hydroxyapatite	Injected around persistent nonhealing extraction socket and adjacent periosteal tissues	Pain reduction, soft tissue healing, consolidated bone formation visible by 6 months	Manimaran et al. [187]
Autologous dental pulp stem cells + BMAC + PRP	Six human patients with mandibular ORN after surgical debridement	β-TCP or HA sponge scaffold + PRP	Local implantation	Improved pain, jaw mobility, oral function, occlusion, and radiographic mandibular regeneration; no major complications during mean 28-month follow-up	Kanakaraj et al. [180]
Platelet-rich fibrin	Human maxillary/mandibular ORN case	Autologous PRF fibrin network	Local placement into surgically debrided ORN site	Mandibular mucosa healed within 3 weeks; radiographic bone healing observed at 10 months	Chen and Chang [189]
Autologous serum-albumin scaffold	Two human patients with refractory stage III mandibular ORN and pathological fracture	Patient-specific cross-linked, lyophilized autologous serum-albumin scaffold	Local implantation after conservative debridement	Progressive bone-density increases, complete mucosal coverage, fistula resolution, pain relief, and restoration of mandibular continuity over 24 months	Gallego et al. [242]
Localized oxygenation	Two patients with advanced mandibular ORN	H-MOD oxygenating dressing containing Hemarina-M101	Local intraoral application	One patient showed initial mucosal improvement followed by progression; the second achieved complete mucosal healing with no exposed bone at 6 months	Duvaud et al. [122]

## 6 Challenges and future directions

ORN remains a major clinical challenge due to its complex, multiscale pathophysiology and its combined detrimental effects on bone, vasculature, immune function, and overlying soft tissues. While RT remains a cornerstone of HNC treatment, the resulting long-term tissue damage underscores the need for therapeutic strategies that address not only structural loss but also persistent biological dysfunction within irradiated tissues. Despite the encouraging preclinical evidence discussed in this article, none of the completed studies directly evaluated senolytic or senomorphic therapy in a mandibular ORN model or in ORN patients. Most animal studies used a single high radiation dose directed toward the femur or lower limb and initiated treatment immediately after irradiation. ORN, in contrast, commonly develops months or years after fractionated HNC RT and involves a complex interaction among cellular senescence, vascular injury, fibrosis, mucosal breakdown, microbial exposure, mechanical trauma, and impaired immune function.

While senotherapeutic agents are promising, they are not specifically approved by the FDA for clinical use to target senescent cells. Although animal studies show strong results and early human trials suggest short-term functional benefits, important questions about proper dosing and off-target cytotoxicity remain unanswered. Future studies should therefore evaluate senotherapeutics in clinically relevant mandibular models using fractionated radiation schedules and delayed treatment after senescence has become established. Outcomes should include not only microCT and senescence markers but also mandibular vascularity, fibrosis, osteocyte viability, mucosal healing, infection, mechanical integrity, bone remodeling, and functional regeneration. Studies should also determine whether senolytic treatment interferes with tumor control or removes senescent cells involved in wound repair and immune surveillance.

Beyond efficacy, the clinical translation of senotherapeutic-biomaterial combination strategies faces substantial regulatory and manufacturing hurdles. Constructs that combine a biomaterial scaffold with senolytics, growth factors, cells, or EVs are likely to be classified as combination products by the FDA, requiring coordination across device, biologic, and drug regulatory frameworks depending on the primary mode of action. This classification complexity increases development timelines and costs, and demands early engagement with regulatory agencies to define the appropriate approval pathway. Manufacturing presents additional challenges: cell- and EV-loaded biomaterials require reproducible, scalable production under good manufacturing practice (GMP) conditions, with rigorous quality control of cell source, passage number, loading efficiency, and release kinetics [138]. Cryopreservation and shelf-life limitations further complicate clinical deployment [185]. For senolytic small molecules, pharmacokinetic challenges including low bioavailability, rapid systemic clearance, and off-target cytotoxicity must be addressed through formulation strategies such as bone-targeted liposomes or controlled-release scaffolds before clinical translation is feasible [236,237]. Taken together, these barriers underscore the need for early-phase clinical trials designed specifically for ORN that incorporate rigorous safety monitoring, standardized outcome measures, and mechanistic endpoints alongside functional assessments.

Future efforts in ORN management should therefore prioritize integrated combination strategies that unite senescence-targeting interventions with bone tissue engineering approaches. Localized delivery of senolytic or senomorphic agents using biomaterial scaffolds or controlled-release systems may allow precise targeting of pathological senescent cell populations while minimizing systemic toxicity. When combined with stem cells, osteoinductive factors, or angiogenic cues, such approaches have the potential to both remove barriers to healing and actively promote restoration of healthy tissue architecture and function.

In summary, the future of ORN treatment lies in interdisciplinary, mechanism-driven approaches that address both the causes and consequences of radiation-induced tissue injury. By integrating senotherapeutic strategies with regenerative medicine, it may be possible to move beyond symptomatic management toward durable biological resolution of ORN, improving functional outcomes and quality of life for patients undergoing RT for HNC.

## 7 Conclusion

ORN should no longer be seen solely as an inexorable consequence of structural tissue destruction, but rather as a pathological state perpetuated by a maladaptive irradiated microenvironment. This conceptual shift reframes treatment from the mere replacement of necrotic tissue toward the restoration of regenerative competence. The integration of senescence-targeted interventions with biomaterial, cellular, and molecular regenerative platforms may therefore represent the most cogent path toward durable recovery by concurrently dismantling the biological impediments to healing and reinstating the mechanisms required for functional bone regeneration.

## CRediT authorship contribution statement

**Aleyna E. La Croix:** Writing – review & editing, Writing – original draft, Visualization, Validation, Methodology, Investigation, Formal analysis, Data curation, Conceptualization. **Maria Eduarda Torres Gouveia:** Writing – review & editing, Writing – original draft, Visualization, Methodology, Investigation, Formal analysis, Data curation, Conceptualization. **Peter M. Bertone:** Writing – review & editing, Writing – original draft, Visualization, Validation, Methodology, Investigation, Formal analysis, Data curation. **Neva M. Nemanic:** Writing – original draft, Investigation. **Chelsea S. Bahney:** Writing – review & editing, Supervision, Conceptualization. **Katherine R. Hixon:** Writing – review & editing, Validation, Supervision, Resources, Project administration, Methodology, Funding acquisition, Formal analysis, Conceptualization.

## Ethics approval and consent to participate

Not applicable. This article is a review and does not involve human participants, animals, or patient data.

## Funding

This work was supported in part by the 10.13039/100000002National Institutes of Health (10.13039/100000002NIH) 10.13039/100000072National Institute of Dental and Craniofacial Research (10.13039/100000072NIDCR) R01DE034025 and the 10.13039/100000002NIH National Institute for Biomedical Imaging and Bioengineering (10.13039/100000070NIBIB) T32 Training in Surgical Innovation Program (T32EB021966-07).

## Declaration of competing interest

The authors declare no competing financial interests or personal relationships that could have influenced the work reported in this paper. K.R. Hixon reports funding from the NIH National Institute of Dental and Craniofacial Research (R01DE034025) and the NIH National Institute for Biomedical Imaging and Bioengineering (T32EB021966-07). No other funding or relationships are declared.
